# A hierarchical Bayesian approach for handling missing classification data

**DOI:** 10.1002/ece3.4927

**Published:** 2019-03-02

**Authors:** Alison C. Ketz, Therese L. Johnson, Mevin B. Hooten, N. Thompson Hobbs

**Affiliations:** ^1^ Natural Resource Ecology Lab Department of Ecosystem Science and Sustainability, and Graduate Degree Program in Ecology Colorado State University Fort Collins Colorado; ^2^ National Park Service Rocky Mountain National Park Estes Park Colorado; ^3^ U.S. Geological Survey Colorado Cooperative Fish and Wildlife Research Unit Colorado State University Fort Collins Colorado; ^4^ Department of Fish, Wildlife and Conservation Biology Colorado State University Fort Collins Colorado; ^5^ Department of Statistics Colorado State University Fort Collins Colorado

**Keywords:** *Cervus elaphus nelsoni*, classification data, demographic ratio, elk, hierarchical Bayesian statistics, missing not at random data, multinomial distribution, proportion estimation, sex ratio, Wildlife Management

## Abstract

1. Ecologists use classifications of individuals in categories to understand composition of populations and communities. These categories might be defined by demographics, functional traits, or species. Assignment of categories is often imperfect, but frequently treated as observations without error. When individuals are observed but not classified, these “partial” observations must be modified to include the missing data mechanism to avoid spurious inference.
2. We developed two hierarchical Bayesian models to overcome the assumption of perfect assignment to mutually exclusive categories in the multinomial distribution of categorical counts, when classifications are missing. These models incorporate auxiliary information to adjust the posterior distributions of the proportions of membership in categories. In one model, we use an empirical Bayes approach, where a subset of data from one year serves as a prior for the missing data the next. In the other approach, we use a small random sample of data within a year to inform the distribution of the missing data.
3. We performed a simulation to show the bias that occurs when partial observations were ignored and demonstrated the altered inference for the estimation of demographic ratios. We applied our models to demographic classifications of elk (*Cervus elaphus nelsoni*) to demonstrate improved inference for the proportions of sex and stage classes.
4. We developed multiple modeling approaches using a generalizable nested multinomial structure to account for partially observed data that were missing not at random for classification counts. Accounting for classification uncertainty is important to accurately understand the composition of populations and communities in ecological studies.

## 1 INTRODUCTION

Understanding the fundamental controls on population dynamics and understanding the consequences of variation in life history theory depend on the interactions of demographic, evolutionary, and ecological forces (Lowe, Kovach, & Allendorf, 2017). Observations of population age and sex composition form the basis for inference on demography, reflecting variation in survival, recruitment, and dispersal processes (Boyce, Haridas, & Lee, 2006; Schindler et al., 2015). These observations are often based on the classification of individuals into demographic categories (Boyce et al., 2006; Koons, Iles, Schaub, & Caswell, 2016), especially when data on individually marked individuals are not available (Koons, Arnold, & Schaub, 2017).

Estimates of demographic parameters and statistics that depend on classification data are frequently used in conservation, monitoring, and adaptive management (Bassar et al., 2010; Lahoz‐Monfort, Guillera‐Arroita, & Hauser, 2014). Sex ratios are used in hunting and fishing regulations because optimal harvest yields depend on age and sex composition (Bender, 2006; Hauser, Cooch, & Lebreton, 2006; Jensen, 1996; Murphy & Smith, 1990). Disease management strategies based on prevalence and transmission rates depend on disease status obtained from imperfect diagnostic testing (PCR, ELISA, visual inspection, etc.) that can have major ramifications for management, particularly for diseases that disproportionately affect subgroups of populations (Hobbs et al., 2015; Lachish & Murray, 2018). Samuel and Storm (2016) corrected age classifications of white‐tailed deer in Wisconsin for models of transmission of chronic wasting disease and found monotonically increasing age‐prevalence patterns and high risk of infection for adult males that were not apparent when the same data were used to estimate prevalence without accounting for age classifications or disease‐associated mortality. Stage‐ or age‐specific survival probabilities obtained from marked populations (Challenger & Schwarz, 2009; Kendall, 2004) are used in structured matrix population models (Caswell, 2001; Skalski, Ryding, & Millspaugh, 2005) and integrated population models (Besbeas, Freeman, Morgan, & Catchpole, 2004; Schaub & Abadi, 2011; Zipkin & Saunders, 2018) to determine population growth rates, and are compromised when life stages and characteristics are difficult to observe (Zipkin & Saunders, 2018). Ketz, Johnson, Monello, and Hobbs (2016) used classification data of elk in Rocky Mountain National Park in an age‐structured integrated population model to obtain demographic parameters when mark–recapture data were unavailable and ignored partial observations that may have influenced model outcomes, which in turn may influence the choice to cull animals to prevent overabundance.

Investigators estimate composition from counts of individuals in categories. Physical characteristics, such as differences in color, size, alternative plumage (Rohwer, 1975), and presence or absence of features such as antlers in ungulates (Smith & McDonald, 2002), are used to differentiate ages, stages, or sex categories. Behavioral differences, including sexual segregation (Bowyer, 2004; Gregory, Lung, Gering, & Swanson, 2009) and alternative auditory song patterns (Volodin, Volodina, Klenova, & Matrosova, 2015), are another method used to classify individuals. Classifications are rarely perfect, creating a need to deal with the uncertainty that arises if only some individuals are classified. Models depend on the assumption of perfectly observed mutually exclusive classifications (Agresti, 2002), which is often unrealistic.

Many species exhibit classification ambiguity, which means that animals may be counted, but cannot be positively classified. As a result, classification data almost always include a category for counts of unclassified individuals. Handling these unknowns has been demonstrably problematic in surveys of aquatic (Cailliet, 2015; Sequeira, Thums, Brooks, & Meekan, 2016; Tsai, Liu, Punt, & Sun, 2015), terrestrial (Boulanger, Gunn, Adamczewski, & Croft, 2011; White, Freddy, Gill, & Ellenberger, 2001), and aerial (Cunningham, Powell, Vrtiska, Stephens, & Walker, 2016; Nadal, Ponz, & Margalida, 2016) species. Classification uncertainty has multiple causes, including physical and behavioral ambiguities, observer skill level, and sampling effort (time). Volunteer participants in ecological surveys are used with increasing frequency (Silvertown, 2009; Swanson et al., 2015). The skill level of an observer can be difficult, if not impossible to assess, because of variation in the knowledge of observers, variability in environmental conditions when observations are made, and differences in observation methods. These uncertainties can be mitigated by using only skilled observers or by specialized training; however, even experts can be unable to completely classify individuals (Conn et al., 2013; Smith & McDonald, 2002).

Conn et al. (2013) describe three general types of observation problems for classification data, including misclassification, partial observation, or both. Misclassification occurs when individuals are assigned to the wrong category, a problem that will not be treated here; for examples in age and stage distributions see Conn and Diefenbach (2007), for mark–recapture see Kendall (2009); Conn and Cooch (2008); Pradel (2005); Kendall (2004); Nichols, Kendall, Hines, and Spendelow (2004), for occupancy models see Ruiz‐Gutierrez, Hooten, and Campbell Grant (2016); Miller et al. (2011); Kendall (2009); Nichols, Hines, Mackenzie, Seamans, and Gutièrrez (2007), and for disease see Jackson, Sharples, Thompson, Duffy, and Couto (2003); Hanks, Hooten, and Baker (2011). In the case of partial observation, individuals are only assigned a category when the observers are certain and the remainder are assigned to an “unknown” category. Partial observations are a form of missing data and can influence model outcomes for structured populations when the age distribution in wildlife populations is not known (Conn & Diefenbach, 2007).

The three types of missing data patterns include missing completely at random, missing at random, and missing not at random (Little & Rubin, 2002; Rubin, 1976). Inference depends upon the missing data mechanism, and how it is accounted for in the model (Nakagawa & Freckleton, 2008). There are several approaches for handling missing data, including ignoring the missing data, data augmentation, and data imputation (Nakagawa & Freckleton, 2008). If the data are missing completely at random, the missing data are a random sample from the distribution of observed values (Bhaskaran & Smeeth, 2014; Heitjan & Basu, 1996). The missing data mechanism has no influence on the outcome of the observations and can be ignored without affecting inference (Little & Rubin, 2002; Rubin, 1976). Missing at random describes the scenario where the missing data may be systematically different from the observed values, but these systematic differences can be completely explained by conditioning on simultaneously observed auxiliary data (Heitjan & Basu, 1996). A typical example is in social or health surveys where questions may be unanswered but could be imputed using other completely observed answers (Agresti & Hitchcock, 2005; Bhaskaran & Smeeth, 2014; Heitjan & Basu, 1996). The extent of the systematic differences and the extent to which they can be recovered by conditioning on the additional data are key to the ignorability of the missing at random mechanism (Bhaskaran & Smeeth, 2014). Missing at random relaxes the strict missing completely at random assumption of unobserved data arising from the identical distribution as observed data, although fundamentally, it is untestable, depends on the unobserved values, and the appropriateness also depends on context (Bhaskaran & Smeeth, 2014). Bayesian models for missing at random data in a multinomial framework (Agresti & Hitchcock, 2005) have been used extensively to impute these non‐ignorable, non‐response data with auxiliary data (Kadane, 1985; Nandram & Choi, 2010). However, in ecology, these data are not necessarily available or relevant, necessitating an alternative approach. The missing data mechanism must be explicit to account for the systematic differences between observed and unobserved values when data are missing not at random. In population ecology, the distributions of ages and sex of individuals within a population do not arise strictly randomly (Krause, Croft, & James, 2007). Observations must account for imperfect detection, particularly when data are missing systematically (Kellner & Swihart, 2014).Treating the data that arise from observations of these systems as completely random, where missing data or incomplete classifications are ignored, can lead to spurious inference of population or community trends.

We use the multinomial distribution to model classification counts and alter the model structure to account for the missing data mechanism. Weak identifiability of the parameters is a fundamental problem for the multinomial distribution and is amplified by flat priors used for the proportions of each level, as is common practice when using the conjugate Dirichlet distribution (Swartz, Haitovsky, Vexler, & Yang, 2004). Introducing additional parameters to account for the non‐ignorable partial observations can exacerbate these identifiability problems; therefore, auxiliary data should be used if possible (Conn & Diefenbach, 2007). We developed two approaches for handling partially observed missing not at random data by explicitly modeling how the missing data mechanism is influencing the observation process. We urge ecologists to incorporate their knowledge of the system into models (Hobbs & Hooten, 2015), even if auxiliary data are unavailable or difficult to obtain, to account for the stages or species that are observed and not classified because of uncertainty.

**Figure 1:**
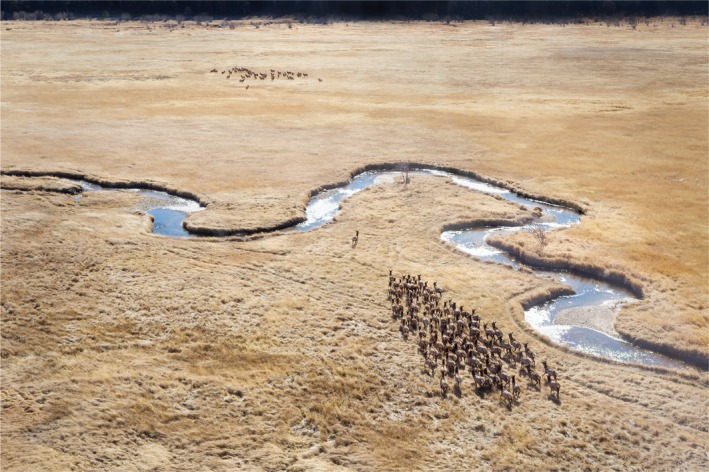
Elk in the winter range of Rocky Mountain National Park. Photograph by Alison Cartwright Ketz (http://www.alisoncartwright.com)

We used simulation to demonstrate the bias that occurs when the missing data mechanism is ignored for partial observations, when data consist of counts of sex and stage classes that are not entirely categorized, and how this bias influenced standard metrics of populations including demographic ratios (Skalski et al., 2005). We developed two modeling approaches to account for the missing data mechanism including an empirical Bayes approach and a small random sub‐sampling routine to provide auxiliary data for the correction of partial observations. We applied these modeling approaches to obtain the posterior distributions of two demographic ratios, consisting of the ratios of juveniles to yearling and adult females, and the ratios of yearling and adult males to females for elk in Rocky Mountain National Park and Estes Park, CO across five winters (Figure 1).

## 2 MATERIALS AND METHODS

### 2.1 Data collection and description

Five years of elk classification data were collected during ground transect surveys on the winter range of Rocky Mountain National Park and in the town of Estes Park, Colorado, from 2012 to 2016. Fifteen independent repeated surveys occurred throughout winter during each year (except twelve surveys the first year). Surveys were executed using volunteer observers who drove road transects and recorded counts of groups that were seen along the transect routes. In addition to overall counts of sighted groups, observers classified individuals into four sex and stage classes consisting of juveniles, yearling males, adult males, yearling, and adult females as well as an additional group of unknown sex or stage.

There was substantial variation among volunteers in their ability to classify elk groups completely. The largest groups were particularly noticeable in that they were most likely to appear in the unknown classification column. Juvenile, yearling, and adult female elk in the Rocky mountains are known to aggregate into large herds in the low‐lying valleys of their ranges during winter (Altmann, 1952). Counting these large groups requires extensive time to obtain an overall count, let alone a classified one. Moreover, it can be difficult to differentiate stages of female elk because they lack the visual cue of antlers. Smith and McDonald (2002) estimated the average discrepancies of classifications for antler‐less elk, consisting of juveniles, yearling, and adult females to be 14%, even for skilled observers, demonstrating the difficulty of obtaining complete classification observations. These data may contain elements of misidentification in addition to partial observations, although we strictly focused on handling the problem of partial observations here.

### 2.2 Model development

We provide two approaches for modeling the data that properly account for uncertainty arising from the unknown classification category, and we present a third approach where we ignore the unknowns to use as a baseline for comparison. We modeled the classification count data (***y***_*t*,*i*_) in *J* = 4 mutually exclusive categories, along with an additional category of unclassified individuals (*z*_*t*,*i*_), during *i* = 1, …, *I*_*t*_ surveys within *t* = 1, …, *T* years (*T* = 5). The likelihood component for these counts was equivalent for all models, although different auxiliary data approaches were used for handling the unclassified counts. In the first model, we used a subset of the classification data from a year of the study to inform the distribution of unclassifieds the following year. In the second model, we used a small random sample of the classified groups to inform the distribution of the unclassifieds within the same year and excluded the random sample subset from the original classification data.

The classification counts including the unknowns were modeled with a multinomial distribution assuming constant proportions of each category across *i* = 1, …, *I*_*t*_ surveys within *t* = 1, …, *T* years, such that

(1) $${\left(\begin{array}{c} y\\ z \end{array}\right)}_{t,i}∼\text{multinomial}\left(N_{t,i},p_{t}\right)$$

where $N_{t,i}=\sum_{j=1}^{J}y_{j,t,i}+z_{t,i}$ is the total observed count of individuals. The vector of proportions (***p***_*t*_) is specified as a function *f* of the true proportions of the *j* = 1, …, *J* classes (***π***_*t*_), the proportion of the unclassified individuals (*p*_*z*,*t*_), and a set of weights ***ω***_*t*_ indicating the probability of the total number of unclassified individuals that should be assigned to each class. Thus,

(2) $$p_{t}=f\left(\pi_{t},p_{z,t},\omega_{t}\right)={\left(\begin{array}{c} \pi -p_{z}\times \omega \\ p_{z} \end{array}\right)}_{t}.$$

See Table 1 for definitions of data vectors and parameters. The unclassified counts (*z*_*t*,*i*_) were modeled with a nested multinomial with the weights (***ω***_*t*_) describing the proportion of the unclassified counts of each of the *J* classes and the constraint $\sum_{j=1}^{J}\omega_{j,t}=1$. For example, observed proportions for each category when *J* = 4 are

(3) $$p_{t}=\left(\begin{array}{c} \pi_{1,t}-\omega_{1,t}\times p_{z,t}\\ \pi_{2,t}-\omega_{2,t}\times p_{z,t}\\ \pi_{3,t}-\omega_{3,t}\times p_{z,t}\\ \pi_{4,t}-\omega_{4,t}\times p_{z,t}\\ p_{z,t} \end{array}\right).$$

**Table 1:** Definitions of sex/stage classes and their corresponding parameters used in the likelihood (Equation 1)

Variable	Proportion	Sex/stage class
*y*_1_	*π*_1_	Juveniles, females and males
*y*_2_	*π*_2_	Yearling and adult females
*y*_3_	*π*_3_	Yearling males
*y*_4_	*π*_4_	Adult males

A Dirichlet prior was used for all proportions across the *T* years, including ***π***_*t*_ and ***ω***_*t*_, and was specified using independent gamma distributions (Gelman, Rubin, Stern, & Garlin, 2014). A uniform prior was used for the unknown category proportions *p*_*z*,*t*_ (Supporting Information Appendix S1). Additional data including environmental covariates or observations to assess sampling effort and expertise of observers were not collected in our study system. Instead, we explicitly altered the model structure to account for the missing data mechanism, rather than relying on informed priors of model parameters.

We assumed that the composition of the unclassified groups would reflect the composition of a subset of the classified groups, based on the sex and stages of the individuals within the classified groups. Sexual segregation is common in vertebrate species (Ruckstuhl & Neuhaus, 2005), particularly for ungulates (Bowyer, 2004), and leads to different compositions of assemblages. Juveniles, yearling and adult females aggregate into large herds during winter, with the occasional presence of very few yearling and adult males. Conversely, yearling and adult male elk form segregated smaller herds or demonstrate solitary behavior (Bowyer, 2004). We assumed that unclassified individuals were likely the result of difficult to distinguish juvenile, yearling, and adult female groups, although it should be noted that yearling and adult males are often present in these large groups albeit in small numbers.

We defined the subset of the data for the *k*th group within survey *i* of the *t*th year, (***x***_*t*,*i*,*k*_), based on the criteria that the sum of the yearling and adult female elk was greater than the sum of the yearling and adult male elk for groups with no unclassified observations ($\sum_{j=1}^{2}y_{j,t,i,k}>\sum_{j=3}^{4}y_{j,t,i,k}$). Although this assumption is highly specific for our study system, our approach is easily altered for other species, particularly because sexual segregation and sexual dimorphism are common (Ruckstuhl & Neuhaus, 2005).

In the first model, we used an empirical Bayesian approach (Gelman et al., 2014), where all subsetted classification data from year *t* (***x***_*t*,*i*,*k*_) were used to predict the posterior distribution of the unknowns the following year (***ω***_*t*+1_). For the first year of the study, we defined a prior for ***ω***_1_ derived from moment matching proportions (Hobbs & Hooten, 2015) based on the mean proportions from Peek and Lovaas (1968) for a winter range area heavily populated by juveniles and adult female elk groups in Montana (***ω***_1_ ~ Dirichlet (23,71,4,2)). The empirical Bayes model for unclassified data was

(4) $$x_{t,i,k}∼\text{multinomial}\left(\sum_{j=1}^{J}x_{j,t,i,k},\omega_{t+1}\right),$$

for the *k*th group in the *i*th survey of the *t* = 2, …, *T* year, with a vague prior on the proportions of the classes (***ω***_*t*+1_ ~ Dirichlet(1,1,1,1)).

In the second model, we used an out‐of‐sample approach where a small random sample of the subsetted auxiliary data, $x_{t,i,k}^{*}$, was used to predict the posterior distributions of the proportions of each of the missing data classes ***ω***_*t*_ within that same year. The sub‐sampled data were removed from the overall data, such that $y_{t,i}^{*}=y_{t,i}-\sum_{k=1}^{K_{t,i}}x_{t,i,k}^{*}$, ensuring that the data were only used once. Thus, the out‐of‐sample model with the nested model for unclassifieds was

(5) $$y_{t,i}^{*}∼\text{multinomial}\left(N_{t,i}^{*},p_{t}\right),$$

(6) $$x_{t,i,k}^{*}∼\text{multinomial}\left(\sum_{j=1}^{J}x_{j,t,i,k}^{*},\omega_{t}\right).$$

For comparison, we modeled the classifications as missing completely at random (hereafter, trim), ignoring the missing data mechanism by omitting *z*_*t*,*i*_ and the nested multinomial from the overall likelihood, given by

(7) $$y_{t,i}∼\text{multinomial}\left(N_{t,i},\pi_{t}\right),$$

for *j* = 1, …, *J* categories, *i* = 1, …, *I*_*t*_ surveys and *t* = 1, …, *T* years, where $N_{i,t}=\sum_{j=1}^{J}y_{j,i,t}$. Full model statements with prior specifications are in Supporting Information Appendix S1.

### 2.3 Model fitting

A simulation was conducted to test the ability of all models to find the posterior distributions of known parameters. The marginal posterior distributions were approximated using Markov chain Monte Carlo (MCMC) using the “dclone” package (Sólymos, 2010) for parallelization of the JAGS software (Plummer, 2003) in R (R Core Team, 2016) (see Supporting Information Appendix S2 for R code and JAGS model statements). Each of the models was fit separately, using three chains consisting of 100,000 MCMC iterations and a burn‐in of 25,000 iterations. Posterior predictive checks indicated no lack of fit, and Gelman‐Rubin diagnostics indicated convergence of all posterior distributions (Gelman et al., 2014). We calculated the difference between the predicted and true proportions of the simulated classes of yearling and adult females (*π*_2,*t*_) because this proportion is used to calculate both demographic ratios (Skalski et al., 2005). For each MCMC iteration, we derived the difference between the predicted values and the true value that was used for generating the data. The empirical Bayes model and the trim model were approximated with varying values of the proportion of unclassified individuals, *p*_*z*_ ∊ {0.1, …, 0.6} to examine the influence of bias when ignoring the proportion of unknowns. We then determined the influence of the out‐of‐sample size on the width of the equal‐tailed Bayesian credible intervals of the proportion of yearling and adult females (*π*_2,*t*_) by repeatedly fitting the out‐of‐sample model for increasing sample sizes of auxiliary data $x_{t,i,k}^{*}$.

The posterior distributions of the proportions of elk in the four sex/stage classifications across 5 years were approximated using all three models (empirical Bayes, out‐of‐sample, and trim). We calculated the posterior distributions of the derived ratios of juveniles to yearling and adult females, as well as the ratios of yearling and adult males to females. For the out‐of‐sample model, we used a sample size of eight observations of the auxiliary data consisting of group level counts within each year, $x_{t,i,k}^{*}$, based on the simulation results. The posterior distributions were obtained using the same MCMC procedures used in the simulation.

## 3 RESULTS

**Figure 2:**
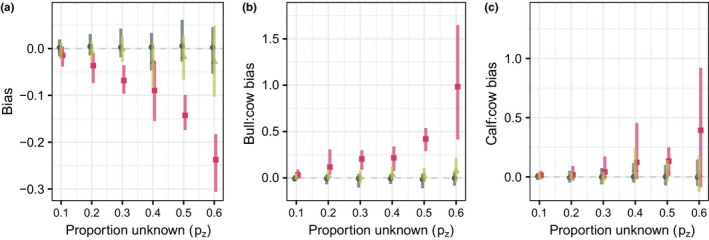
(a) The posterior distributions of the difference between the generated proportion of yearling and adult females (*π*_2_) and the true value for the empirical Bayes approach (black squares), out‐of‐sample approach (yellow triangles), and ignoring the unclassified data with the trim approach (red circles), for increasing proportions of missing unclassified data (*p*_*z*_). Bias increases as missing data increases and is ignored, for the juvenile to yearling and adult female ratio (b) and for the ratio of yearling and adult males to yearling and adult females (c)

**Figure 3:**
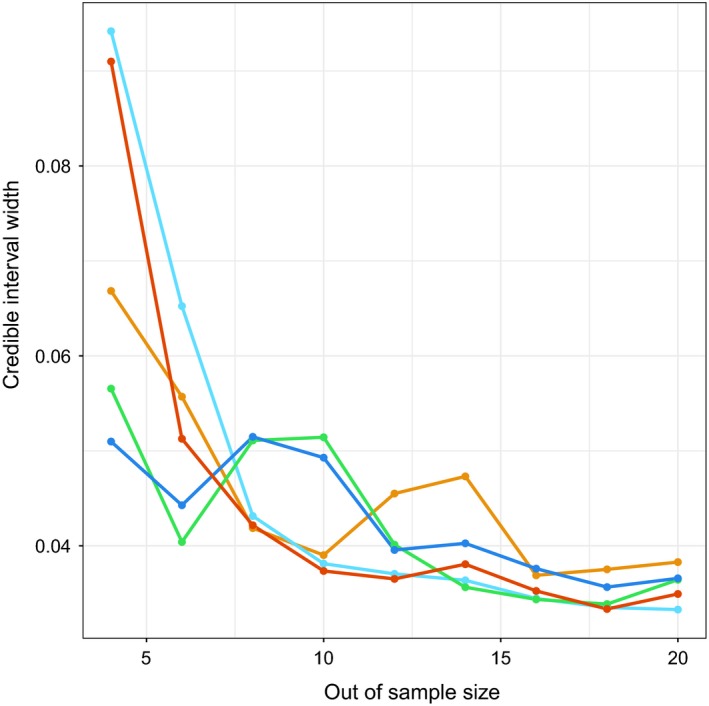
The equal‐tailed 95% Bayesian credible interval width of the proportion of yearling and adult females (*π*_2_) in the simulation, for year 1 (orange), year 2 (light blue), year 3 (green), year 4 (dark blue), and year 5 (red) decreased as the size of the out‐of‐sample subset of data increased

Simulation results indicated that an increasing proportion of unclassified individuals (*p*_*z*_) amplified the bias of the proportion of yearling and adult females (Figure 2a) when unknowns were ignored. Both of the demographic ratios were overestimated, including the ratio of juveniles to yearling and adult females (Figure 2b), and the ratio of yearling and adult males to yearling and adult females (Figure 2c). Simulation results testing the out‐of‐sample model across values of *p*_*z*_ indicated that the equal‐tailed 95% Bayesian credible interval width decreased as the out‐of‐sample size increased, until approximately 8–10 samples, after which very little change occurred for the credible interval width (Figure 3). As the out‐of‐sample size increased, there was no effect on the bias when the proportion of partially observed groups (*p*_*z*_) remained constant (Supporting Information Appendix S3, Figure S2).

**Figure 4:**
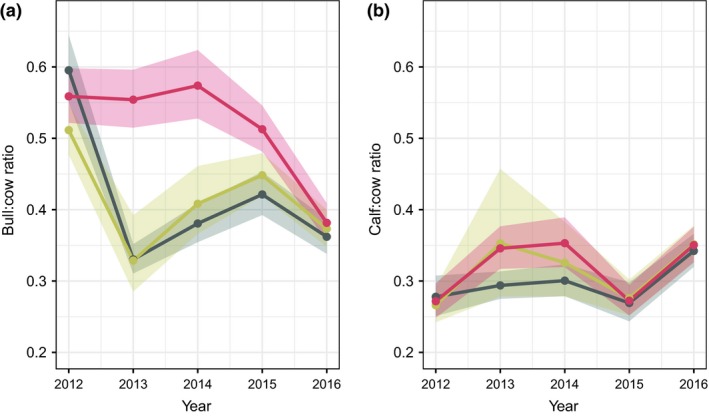
The marginal posterior distributions for (a) the ratio of yearling and adult males to yearling and adult females and (b) the ratio of juveniles to yearling and adult females, from 2012 through 2016, using the medians (gray circles) of the empirical Bayes model with equal‐tailed 95% Bayesian credible intervals (gray shaded region), medians of the out‐of‐sample model (yellow circles) and Bayesian credible intervals (yellow shaded region), and medians of the trim model (red circles) and Bayesian credible intervals (red shaded region)

The medians of the marginal posterior distributions of the proportion of yearling and adult females for elk in Rocky Mountain National Park (*π*_2_) were similar for the empirical Bayes and out‐of‐sample models, although differed substantially from the trim model (Table 2 and Supporting Information Appendix S4) for 3 of the 5 years. The empirical Bayes and out‐of‐sample models had nearly completely overlapping marginal posterior distributions of the ratios of juveniles to yearling and adult females ($\pi_{1}/\pi_{2}$) throughout the years (Figure 4b) and for the ratio of yearling and adult males to females ($\left(\pi_{3}+\pi_{4}\right)/\pi_{2}$) (Figure 4a). The posterior distributions for the yearling and adult males to females ratios under both proposed models were substantially different from the posterior distributions of the trim model.

**Table 2:** Medians of the posterior distributions of the proportions of yearling and adult females (*π*_2_) from 2012 through 2016 for elk in Rocky Mountain National Park derived from three models including the empirical Bayes approach (EBA), out‐of‐sample (OOS), and ignoring (Trim) approaches

Year	EBA	OOS	Trim
2012	0.53	0.56	0.52
2013	0.62	0.62	0.61
2014	0.59	0.58	0.47
2015	0.59	0.60	0.51
2016	0.59	0.58	0.58

**Figure 5:**
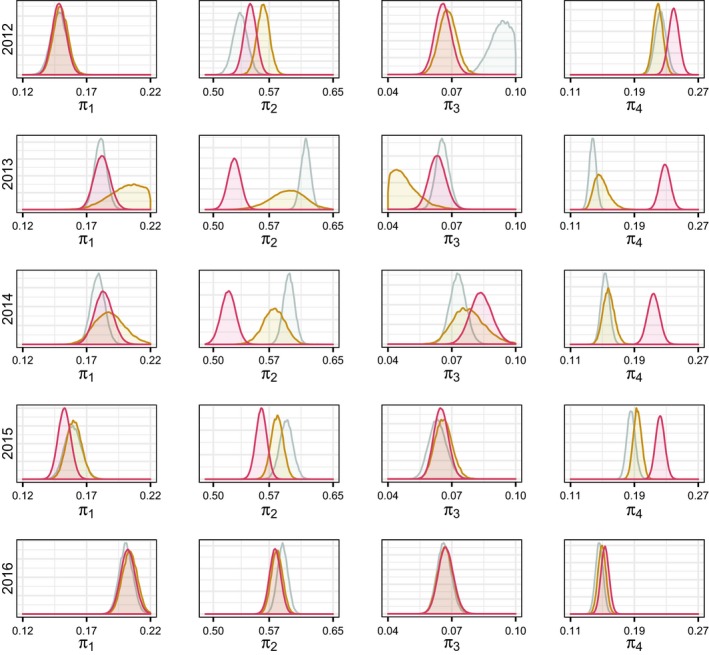
The densities of the marginal posterior distributions for the proportions of each stage/sex classes including juveniles (*π*_1_), yearling and adult females (*π*_2_), yearling males (*π*_3_), and adult males (*π*_4_) from 2012 through 2016, using the empirical Bayesian approach (gray), out‐of‐sample approach (yellow), and the trim model ignoring the unclassified data (red)

The posterior distributions for the proportions of yearling and adult females (*π*_2,*t*_) and proportions of adult males (*π*_4_) across all years of the study demonstrated the altered inference that occurred when the partial observations were accounted for in the model (Figure 5). For three of the years, the posterior distributions of the proportion of adult males were nearly identical for the empirical Bayes and out‐of‐sample models, but with no overlap of the trim model, suggesting that the bias that occurs when ignoring the unclassified data greatly alters inference.

## 4 DISCUSSION

Properly estimating the composition of populations and communities using counts of individuals assigned to categories forms a frequent challenge in ecological research. Uncertainty in classification data commonly arises because individuals are counted but not classified, producing an “unknown” category. Correcting for bias that can result from falsely assuming that this unknown category is proportionally the same as the knowns is critical if these data are to be used for fitting demographic models (Conn et al., 2013).

Simulation results demonstrated the increasing bias that occurred as the number of unknown individuals increased when these observations were ignored (Figure 2). The result is intuitive, but would not have occurred if the data had been missing completely at random and treated as such. We found that the proportion of yearling and adult females (*π*_2_) was underestimated when unknowns were ignored (Figure 2). This finding, in turn, led to overestimation of sex and stage ratios. We used the simulation to determine the number of samples required for an out‐of‐sample approach, where a small subset of observations were used to estimate the proportions of the unknown counts (Figure 2a). Calculating the minimum sample size for a multinomial model depends on several factors, including the number of categories and the values of the proportions of each of the categories (Thompson, 1987). Simulation is useful for determining the minimum sample size to account for these factors. Results suggested that, in our study system, after observing approximately 8–10 groups (Figure 3), the width of the Bayesian credible interval no longer decreased substantially. We chose an out‐of‐sample size of 8, to use the greatest possible proportion of the data in the likelihood. Simulations showed that the empirical Bayes model provided the most accurate bias adjustment for the posterior distributions of the proportion of yearling and adult females (Supporting Information Appendix S3, Figure S1). The out‐of‐sample model was able to recover parameters, but the credible intervals of the marginal posterior distributions of yearling and adult female proportions were less centered around the true parameter values, although many of the credible intervals were able to capture them.

The results of our case study showed little difference in the posterior distributions for the empirical Bayes and out‐of‐sample models, but the proportions of adults of both sexes were substantially different from the trim model (Figure 5). This suggests that there may be no difference among years for the distribution of juvenile, yearling, and adult female groups, which calls into question the assumption of a time‐varying composition explicit in the empirical Bayes model. However, it could also mean that both models adequately adjust for the bias resulting from ignoring partial classifications.

There are several statistical problems that occur in observational studies, including measurement, sampling, and estimation bias (Krebs, 1999). Measurement bias is due to faulty devices or procedures and sampling bias occurs when a sample is not representative of the target population (Walther & Moore, 2005). In both of these circumstances, observations are systematically biased away from the true value, and increasing sampling effort cannot account for these biases because the observations are not a random sample from the population of interest (Walther & Moore, 2005). The posterior distributions of the proportions of the sex and stage classes reflect a type of measurement error that we can explicitly account for, provided that the mechanisms driving that measurement error are assumed known. The empirical Bayes and out‐of‐sample models use model structure and data manipulation to account for bias induced by measurement error that would otherwise be ignored. Estimation bias is another kind of systematic error and could decrease with increasing sample effort (Walther & Moore, 2005). The variability of the classification counts may be susceptible to fluctuations in the presence and detectability of individuals that are available to sample during the transect surveys (Ketz et al., 2018). The proportions of the sex and stage classes (***π***), as well as the classification weights (***ω***), varied by year but were assumed constant within years. Timing of the surveys relative to fluctuations in the spatial distribution of elk in the Estes Park region could drive some of the differences in the demographic ratios (Figure 4). Additional surveys within years or modeling the surveys in a nested structure could potentially improve accuracy and precision by reducing the sampling bias arising from possible violations of the assumption of spatial and temporal closure within years.

We made the critical assumption that the unclassified data arose from groups of juvenile, yearling, and adult females because yearling and adult males can be easily identified during winter based on their antlers (Smith & McDonald, 2002), which was used to overcome the missing not at random mechanism in the model structure. Although this particular assumption is highly specific for elk, there are numerous examples of other species where ecologists could apply similar knowledge of the biology of the species, to subset the data for estimating the proportions in the nested multinomial models that we developed. Bighorn sheep (*Ovis canadensis*) in Colorado illustrate a similar classification problem, because juvenile, yearling, and adult females aggregate and are difficult to differentiate (George, Kahn, Miller, & Watkins, 2009). Another example includes fall surveys of white‐tailed ptarmigan, where approximately 20% of observed individuals cannot be classified because the ptarmigan have not yet molted, so identification of sex is impossible for these individuals (Wann, Aldridge, & Braun, 2014). Classification data from spring surveys when birds are captured and classifiable could be used to adjust fall survey demographic ratios essential for setting hunter harvest regulations.

Both of the proposed models that account for the missing data mechanism have strengths and weaknesses that could be exploited for different study systems. Empirical Bayesian methods are typically criticized for using the data twice and for assuming exchangability (Gelman, 2008). However, for rare or difficult to detect species, empirical Bayes would be a better choice than the out‐of‐sample model because all of the data collected are used in the data observation likelihood. For species that are neither rare nor difficult to detect, the out‐of‐sample model avoids using the data twice with little loss of information.

Identifiability problems can arise for multinomial models, but these can be mitigated by using informed priors and incorporating biological knowledge of the study system (Swartz et al., 2004). It is essential to have auxiliary data, or at the very least, auxiliary information that can be used to obtain the distribution of unknown partially classified data. The way that these data are incorporated into the model structure is highly system and circumstance dependent, but we consider several active areas of ecological analyses where these could be used. For example, camera traps are increasingly used to identify the age, sex, and reproductive processes of populations, and observations may result in unclassified individuals (Gardner, Reppucci, Lucherini, & Royle, 2010). Auxiliary data, such as spatial location of the cameras, could provide information about these unclassified cases similar to leveraging geographic information in spatial capture–recapture models (Royle, Karanth, Gopalaswamy, & Kumar, 2009). Data on genetics implying susceptibility to infection risk or information about biological patterns of disease progression are additional examples of auxiliary data that can be used to inform priors or model structure to account for uncertain disease status resulting from unreliable diagnostic tests (Choi et al., 2009; Haneuse & Wakefield, 2008; Tullman, 2013). Environmental covariates have been used extensively as auxiliary data in capture—recapture analyses coupled with assumptions of temporal, spatial, and individual variation to determine survival and detection probabilities (Pollock, 2002). Walsh, Norton, Storm, Van Deelen, and Heisey (2017) provide a suggestion for auxiliary data consisting of expert opinion to account for uncertainty in cause‐specific survival analysis, when causes of death are unclear. Auxiliary data are increasingly used because of advances in integrated modeling approaches, when multiple data sources can be exploited to improve inference (Luo et al., 2009; Schaub & Abadi, 2011; Warton et al., 2015).

One of the fundamental assumptions of the multinomial distribution is that the outcomes of each event are mutually exclusive and all inclusive (Agresti, 2002). In this paper, we developed a nested multinomial distribution to improve inference for circumstances when this assumption is violated. We improved the inference of the proportions of four sex/stage classes of elk on the winter range of Rocky Mountain National Park and Estes Park, CO (Figure 5), and in turn, we were able to improve inference for demographic ratios used by wildlife managers. Our approach could be applied to a broad variety of ecological applications, where uncertainty about characteristics obscures inference for population, disease, community, and ecosystem ecology.

## CONFLICT OF INTEREST

None declared.

## AUTHOR CONTRIBUTIONS

AK, TH, TJ, and MH substantially contributed to the conception and design of the work. AK and TJ contributed to the acquisition of data. AK, TH, and MH contributed to analysis and interpretation of the data. All authors contributed to reviewing the work for important intellectual content.

## Data Availability

All data supporting this document are available in the Dryad data repository at https://doi.org/10.5061/dryad.8h36t01.
