# Supplementary material for: A hierarchical Bayesian approach for handling missing classification data
Source: Ecol Evol. 2019 Mar 2;9(6):3130–40. doi: 10.1002/ece3.4927 (PMC6434567; doi:10.1002/ece3.4927)
Supplement: Supplementary file 1 [file ECE3-9-3130-s001.pdf]

Appendix S1 - Model Statements

The classification counts were modeled for $i = 1, ..., I_t$ surveys within $t = 1, ..., T$ years, where $N_{i,t} = \sum_{j=1}^{J+1} y_{j,i,t}$, such that

$$\boldsymbol{y}_{i,t} \sim \text{multinomial}(N_{i,t}, \boldsymbol{p}_t). \tag{S1}$$

The vector of proportions ($\boldsymbol{p}_t$) includes the proportions for the $j = 1, ..., J$ classes ($\boldsymbol{\pi}_t$) and the proportion of the unclassified individuals, $p_{z,t}$. Unclassified counts were modeled with proportions ($\boldsymbol{\omega}_t$) describing the J classes and the constraint $\sum_{j=1}^{J} \omega_{j,t} = 1$. Observed proportions for each category when $J = 4$ are

$$\boldsymbol{p}_t = \begin{pmatrix} \pi_{1,t} - \omega_{1,t} \times p_{\text{z},t} \\ \pi_{2,t} - \omega_{2,t} \times p_{\text{z},t} \\ \pi_{3,t} - \omega_{3,t} \times p_{\text{z},t} \\ \pi_{4,t} - \omega_{4,t} \times p_{\text{z},t} \\ p_{\text{z},t} \end{pmatrix}. \tag{S2}$$

$$\boldsymbol{\pi}_t \sim \text{Dirichlet}(\boldsymbol{\alpha}_t) \tag{S3}$$

$$\boldsymbol{\alpha}_t = \boldsymbol{\alpha}*_t / \alpha_0 \tag{S4}$$

$$\alpha_0 = \sum_{j=1}^{J} \alpha*_j \tag{S5}$$

$$\alpha*_j \sim \text{gamma}(.001, .001) \tag{S6}$$

$$p_{\text{z},t} \sim \text{uniform}(0, 1). \tag{S7}$$

S1 Empirical Bayes model

Unclassified data were modeled using juvenile, and adult and yearling female herd count data ($\boldsymbol{x}_{k_t,t}$):

$$\boldsymbol{x}_{k_t} \sim \text{multinomial}(\sum_{j=1}^{J} x_{j,k,t}, \boldsymbol{\omega}_{t+1}), \quad \text{(S8)}$$

$$\boldsymbol{\omega}_1 \sim \text{Dirichlet}(23, 71, 4, 2), \quad \text{(S9)}$$

$$\boldsymbol{\omega}_{t+1} \sim \text{Dirichlet}(1, 1, 1, 1), \quad \text{(S10)}$$

for the kth group in the $t = 2, ..., T$ year. The prior for the first year of the model weights ($\boldsymbol{\omega}_1$) was derived from results of sex and stage proportions in Peek & Lovaas (1968).

S2 Out-of-sample model

Unclassified data were modeled using a small subset of the juvenile, and adult and yearling female herd count data ($\boldsymbol{x}_{k_t,t}$) and these counts were removed from the overall data such that $\boldsymbol{y}^*_{i,t} = \boldsymbol{y}_{i,t} - \sum_{k_{i,t}} \boldsymbol{x}_{t,i_t,k_{i,t}}$, and

$$\boldsymbol{y}^*_{i,t} \sim \text{ multinomial}(N^*_{i,t}, \boldsymbol{p}_t), \quad \text{(S11)}$$

where $N^*_{i,t} = \sum_{j=1}^{J+1} y^*_{j,i,t}$. The nested model for the unclassifieds was

$$\boldsymbol{x}_{k,t} \sim \text{multinomial}(\sum_{j=1}^{J} x_{j,k,t}, \boldsymbol{\omega}_t), \quad \text{(S12)}$$

$$\boldsymbol{\omega}_t \sim \text{Dirichlet}(1, 1, 1, 1). \quad \text{(S13)}$$

S3 Trim model - ignoring unknown category

$$\boldsymbol{y}_{i,t} \sim \text{multinomial}(N_{i,t}, \boldsymbol{\pi}_t), \tag{S14}$$

$$\boldsymbol{\pi}_t \sim \text{Dirichlet}(\boldsymbol{\alpha}_t), \tag{S15}$$

$$\boldsymbol{\alpha}_t = \boldsymbol{\alpha}*_t/\alpha_0, \tag{S16}$$

$$\alpha_0 = \sum_{j=1}^{J} \alpha*_j, \tag{S17}$$

$$\alpha*_j \sim \text{gamma}(.001, .001) \tag{S18}$$

for $j = 1, ..., J$ categories, $i = 1, ..., I_t$ surveys and $t = 1, ..., T$ years, where $N_{i,t} = \sum_{j=1}^{J} y_{j,i,t}$.

S4 Full posterior and joint distributions

We combine all components of the model into the full posterior and joint distributions for $j = 1, ..., J$ categories, $i = 1, ..., I_t$ surveys and $t = 1, ..., T$ years for the empirical Bayes model.

$$[\boldsymbol{\Pi}, \boldsymbol{\alpha}^*, \boldsymbol{p}_z, \boldsymbol{\Omega} | \boldsymbol{Y}, \boldsymbol{X}] \propto \prod_{t=2}^{T} \prod_{i=1}^{I_t} [y_{i,t} | \boldsymbol{\pi}_t, p_z, \boldsymbol{\omega}_{t-1}] \prod_{k=1}^{K_t} [\boldsymbol{x}_k | \boldsymbol{\omega}_t] \times \tag{S19}$$

$$[\boldsymbol{\omega}_t] \left[\boldsymbol{\pi} | \boldsymbol{\alpha}^*\right] \left[\boldsymbol{\alpha}^*\right] [\boldsymbol{p}_z] \times \tag{S20}$$

$$\left[\boldsymbol{y}_{i,1} | \boldsymbol{\alpha}_1^*\right] [\boldsymbol{x}_{k_1} | \boldsymbol{\omega}_1] [\boldsymbol{\omega}_1] \tag{S21}$$

References

Peek, J.M. & Lovaas, A.L. (1968) Differential distribution of elk by sex and age on the Gallatin winter range, Montana. *The Journal of Wildlife Management* **32**, 553–557.
