# Supplementary material for: A hierarchical Bayesian approach for handling missing classification data
Source: Ecol Evol. 2019 Mar 2;9(6):3130–40. doi: 10.1002/ece3.4927 (PMC6434567; doi:10.1002/ece3.4927)
Supplement: Supplementary file 2 [file ECE3-9-3130-s002.pdf]

Appendix S2 - R code and JAGS Model Statements

Data can be downloaded from the dryad repository.

S1 Empirical Bayes model

```
EBA.model=function(){

  #Priors
  #Initial year
  pz[1] ~ dbeta(1,1)
  omega[1,] ~ ddirch(omega.prior)
  pis.temp[1,1:4] <- pis[1,] - pz[1]*omega[1,]
  pis.temp[1,5] <- pz[1]

  for(j in 1:4){
    alpha.star[1,j] ~ dgamma(.001,.001)
    alpha[1,j] <- alpha.star[1,j]/alpha0[1]
  }
  alpha0[1] <- sum(alpha.star[,1])

  pis[1,] ~ ddirch(alpha[1,])

  for(t in 2:T){
    pz[t] ~ dbeta(1,1)
    omega[t,] <- delta[t-1,]
    pis.temp[t,1:4] <- pis[t,] - pz[t]*omega[t,]
    pis.temp[t,5] <- pz[t]

    for(j in 1:4){
      alpha.star[t,j] ~ dgamma(.001,.001)
      alpha[t,j] <- alpha.star[t,j]/alpha0[t]
    }
    alpha0[t]<-sum(alpha.star[t,])

    pis[t,] ~ ddirch(alpha[t,])
  }

  #Likelihood for counts including unknowns
  for(t in 1:T){
    for(i in 1:I[t]){
      Y[t,i,] ~ dmulti(pis.temp[t,],N[t,i])
    }
  }

  #Likelihood for transect level proportion of calf cow herds
  for(t in 1:T){
```

```
    for(k in 1:K[t]){
      X[t,k,] ~ dmulti(delta[t,],Nx[t,k])
    }
    delta[t,] ~ ddirch(delta.prior)
  }

  #Derived parameters
  #calf:cow ratio
  #bull:cow ratio
  for(t in 1:T){
    calfcow[t]<-pis[t,1]/pis[t,2]
    bullcow[t]<-(pis[t,3]+pis[t,4])/pis[t,2]
  }

}#end model
```

S2 Out-of-sample model

```
OOS.model=function(){

  #Priors
  #Initial year
  pz[1] ~ dbeta(1,1)
  pis.temp[1,1:4] <- pis[1,] - pz[1]*omega[1,]
  pis.temp[1,5] <- pz[1]
  for(j in 1:4){
    alpha.star[1,j] ~ dgamma(.001,.001)
    alpha[1,j] <- alpha.star[1,j]/alpha0[1]
  }
  alpha0[1] <- sum(alpha.star[,1])
  pis[1,] ~ ddirch(alpha[1,])

  for(t in 2:T){
    pz[t] ~ dbeta(1,1)
    pis.temp[t,1:4] <- pis[t,] - pz[t]*omega[t,]
    pis.temp[t,5] <- pz[t]

    for(j in 1:4){
      alpha.star[t,j] ~ dgamma(.001,.001)
      alpha[t,j] <- alpha.star[t,j]/alpha0[t]
    }
    alpha0[t]<-sum(alpha.star[t,])

    pis[t,] ~ ddirch(alpha[t,])
  }
```

```
  #Likelihood for counts including unknowns
  for(t in 1:T){
    for(i in 1:I[t]){
      Y[t,i,] ~ dmulti(pis.temp[t,],N[t,i])
    }
  }

  #Likelihood for transect level proportion of calf cow herds
  for(t in 1:T){
    for(k in 1:K){
      X[t,k,] ~ dmulti(omega[t,],Nx[t,k])
    }
    omega[t,] ~ ddirch(omega.prior)
  }

  #Derived parameters
  #calf:cow ratio
  #bull:cow ratio
  for(t in 1:T){
    calfcow[t]<-pis[t,1]/pis[t,2]
    bullcow[t]<-(pis[t,3]+pis[t,4])/pis[t,2]
  }

}#end model
```

S3 Trim model - Ignoring Unknown Category

```
TRIM.model=function(){

  #Priors
  for(t in 1:T){
    for(j in 1:J){
      alpha.star[t,j] ~ dgamma(.001,.001)
      alpha[t,j] <- alpha.star[t,j]/alpha0[t]
    }
    alpha0[t]<-sum(alpha.star[t,])
    pis[t,] ~ ddirch(alpha[t,])
  }

  #Likelihood for counts including unknowns
  for(t in 1:T){
    for(i in 1:I[t]){
      Y[t,i,] ~ dmulti(pis[t,],N[t,i])
    }
  }

  #Derived parameters
```

```
  #calf:cow ratio
  #bull:cow ratio
  for(t in 1:T){
    calfcow[t]<-pis[t,1]/pis[t,2]
    bullcow[t]<-(pis[t,3]+pis[t,4])/pis[t,2]
  }

}#end model
```

S4 Simulation Functions

S4.1 Proportion missing data

The following function generates data, then fits all three models, calculates bias and outputs model results. The function depends on the proportion of missing data (p_z), which must be a real number between 0 and 1.

```
modelrun_pz=function(pzset=.2){

     ###
     ### Function to run EBA/OOS/TRIM models for different values of pz
     ###

    #check that pzset is valid value
    if(pzset>1|pzset<0){
      return("Please enter a value for pz between 0 and 1");
      break;}

    T=5 #number years
    I=c(12,rep(15,4)) #number of surveys in each year
    Imax=max(I) #max number of surveys
    J=4 #number classes

    #set omegas
    omega=matrix(NA,T,J)
    omega[1,]=c(.15,.75,.03,.07)
    omega[2,]=c(.15,.75,.05,.05)
    omega[3,]=c(.2,.71,.03,.04)
    omega[4,]=c(.17,.73,.05,.05)
    omega[5,]=c(.17,.73,.05,.05)

    #set pz
    pz = rep(pzset,T)
```

```
#set pis
pis.true = rdirichlet(T,c(30,100,20,20))

# generate pis.obs
pis.obs=matrix(NA,T,J+1)
for(t in 1:T){
    pis.obs[t,] = c((pis.true[t,] - pz[t]*omega[t,]),pz[t])
}

#Derived parameters
#calf:cow ratio
#bull:cow ratio
calfcow.true=pis.true[,1]/pis.true[,2]
bullcow.true=(pis.true[,3]+pis.true[,4])/pis.true[,2]

#Generate observed proportion in each category, including missing data
N = matrix(NA,T,Imax)
Y = array(NA,c(T,Imax,(J+1)))
#set the number of herds seen within each year within each survey
K.herd = matrix(NA,T,Imax)
for(t in 1:T){
    for(i in 1:I[t]){
        N[t,i] = rpois(1,400)
        Y[t,i,]=rmultinom(1,size=N[t,i],pis.obs[t,])
        K.herd[t,i]=rpois(1,10)+1
    }
}

#Generate X's from the omega's, using a part of the Y's
X.herd=array(NA,c(T,Imax,max(K.herd,na.rm=TRUE),J))
for(t in 1:T){
    for(i in 1:I[t]){
        herds.sum=sum(Y[t,i,1:J])
        herds.indx=sort(sample(1:herds.sum,K.herd[t,i],replace=FALSE))
        N.herd=c(herds.indx[1],diff(herds.indx))
        for (k in 1:K.herd[t,i]){
            X.herd[t,i,k,]=rmultinom(1,N.herd[k],prob=omega[t,])
        }

    }
}
K=apply(K.herd,1,sum,na.rm=TRUE)
Kmax=max(K)

###
### X -- using all calf/cow groups
###
```

```
X.yr1=X.herd[1,1,,]
for(i in 2:I[1]){
    X.yr1=rbind(X.yr1,X.herd[1,i,,])
}
X.yr1=X.yr1[!is.na(X.yr1[,1]),]

X.yr2=X.herd[2,1,,]
for(i in 2:I[2]){
    X.yr2=rbind(X.yr2,X.herd[2,i,,])
}
X.yr2=X.yr2[!is.na(X.yr2[,1]),]

X.yr3=X.herd[3,1,,]
for(i in 2:I[3]){
    X.yr3=rbind(X.yr3,X.herd[3,i,,])
}
X.yr3=X.yr3[!is.na(X.yr3[,1]),]

X.yr4=X.herd[4,1,,]
for(i in 2:I[4]){
    X.yr4=rbind(X.yr4,X.herd[4,i,,])
}
X.yr4=X.yr4[!is.na(X.yr4[,1]),]

X.yr5=X.herd[5,1,,]
for(i in 2:I[5]){
    X.yr5=rbind(X.yr5,X.herd[5,i,,])
}
X.yr5=X.yr5[!is.na(X.yr5[,1]),]

X=array(NA,c(T,Kmax,J))
#Year 1
for(k in 1:K[1]){
    for(j in 1:J){
        X[1,k,j]=X.yr1[k,j]
    }
}
#Year 2
for(k in 1:K[2]){
    for(j in 1:J){
        X[2,k,j]=X.yr2[k,j]
    }
}
#Year 3
for(k in 1:K[3]){
    for(j in 1:J){
```

```
        X[3,k,j]=X.yr3[k,j]
    }
}
#Year 4
for(k in 1:K[4]){
    for(j in 1:J){
        X[4,k,j]=X.yr4[k,j]
    }
}
#Year 5
for(k in 1:K[5]){
    for(j in 1:J){
        X[5,k,j]=X.yr5[k,j]
    }
}

#prior for omega for the first year model EBA and OOS
EBA.omega.prior=c(5,20,1,1)
OOS.omega.prior=c(1,1,1,1)

Nx = matrix(NA,T,Kmax)
for(t in 1:T){
    Nx[t,]=apply(X[t,,],1,sum)
}

delta.prior=c(1,1,1,1)

###
### X.oos -- out of sample
###

num.sample=10
indx.sample=matrix(NA,T,num.sample)
X.oos=array(NA,c(T,num.sample,J))
Y.star=Y
for(t in 1:T){
    indx.sample[t,]=sort(sample(1:I[t],size=num.sample,replace = TRUE))
    X.oos[t,1,]=X.herd[t,indx.sample[t,1],1,]
    Y.star[t,indx.sample[t,1],1:J]=Y.star[t,indx.sample[t,1],1:J]-X.herd[t,indx

    m=1
    for (h in 2:num.sample){
        if(indx.sample[t,h]==indx.sample[t,h-1]){m=m+1}
        X.oos[t,h,]=X.herd[t,indx.sample[t,h],m,]
        Y.star[t,indx.sample[t,h],1:J]=Y.star[t,indx.sample[t,h],1:J]-X.herd[t,
        m=1
    }
```

```
}

Nx.oos=matrix(NA,T,num.sample)
N.star = matrix(NA,T,Imax)
for(t in 1:T){
    Nx.oos[t,]=apply(X.oos[t,,],1,sum)
    N.star[t,] = apply(Y.star[t,,],1,sum)
}

#Ensuring the random sample of Y herds are less than the total, ensuring that Y
while(length(which(Y.star<0))>0){

    num.sample=10
    indx.sample=matrix(NA,T,num.sample)
    X.oos=array(NA,c(T,num.sample,J))
    Y.star=Y
    for(t in 1:T){
        indx.sample[t,]=sort(sample(1:I[t],size=num.sample,replace = TRUE))
        X.oos[t,1,]=X.herd[t,indx.sample[t,1],1,]
        Y.star[t,indx.sample[t,1],1:J]=Y.star[t,indx.sample[t,1],1:J]-X.herd[t,

        m=1
        for (h in 2:num.sample){
            if(indx.sample[t,h]==indx.sample[t,h-1]){m=m+1}
            X.oos[t,h,]=X.herd[t,indx.sample[t,h],m,]
            Y.star[t,indx.sample[t,h],1:J]=Y.star[t,indx.sample[t,h],1:J]-X.her
            m=1
        }
    }
    Nx.oos=matrix(NA,T,num.sample)
    N.star = matrix(NA,T,Imax)
    for(t in 1:T){
        Nx.oos[t,]=apply(X.oos[t,,],1,sum)
        N.star[t,] = apply(Y.star[t,,],1,sum)
    }
}

#################
### EBA model ###
#################

EBA.model=function(){

    #initial year
    pz[1] ~ dbeta(1,1)
    omega[1,] ~ ddirch(omega.prior)
    pis.temp[1,1:4] <- pis[1,] - pz[1]*omega[1,]
```

```
pis.temp[1,5] <- pz[1]

for(j in 1:4){
    alpha.star[1,j] ~ dgamma(.001,.001)
    alpha[1,j] <- alpha.star[1,j]/alpha0[1]
}
alpha0[1] <- sum(alpha.star[,1])

pis[1,] ~ ddirch(alpha[1,])

for(t in 2:T){
    pz[t] ~ dbeta(1,1)
    omega[t,] <- delta[t-1,]
    pis.temp[t,1:4] <- pis[t,] - pz[t]*omega[t,]
    pis.temp[t,5] <- pz[t]

    for(j in 1:4){
        alpha.star[t,j] ~ dgamma(.001,.001)
        alpha[t,j] <- alpha.star[t,j]/alpha0[t]
    }
    alpha0[t]<-sum(alpha.star[t,])

    pis[t,] ~ ddirch(alpha[t,])
}

#Likelihood for counts including unknowns
for(t in 1:T){
    for(i in 1:I[t]){
        Y[t,i,] ~ dmulti(pis.temp[t,],N[t,i])
    }
}

#Likelihood for transect level proportion of calf cow herds
for(t in 1:T){
    for(k in 1:K[t]){
        X[t,k,] ~ dmulti(delta[t,],Nx[t,k])
    }
    delta[t,] ~ ddirch(delta.prior)
}

#Derived parameters
#calf:cow ratio
#bull:cow ratio
for(t in 1:T){
    calfcow[t]<-pis[t,1]/pis[t,2]
    bullcow[t]<-(pis[t,3]+pis[t,4])/pis[t,2]
}
```

```
}#end model

EBA.data=list(pis.temp=matrix(NA,T,J+1),
    pis=matrix(NA,T,J),
    omega=matrix(NA,T,J),
    I=I,
    T=T,
    Y=Y,
    N=N,
    omega.prior=EBA.omega.prior,
    X=X,
    Nx=Nx,
    delta=matrix(NA,T,J),
    delta.prior=c(1,1,1,1),
    K=K,
    pz=rep(NA,T)
    )

EBA.initial=list(
    list(pz=rep(.1,T),alpha.star=matrix(.25,T,J)),
    list(pz=rep(.3,T),alpha.star=matrix(rep(c(.4,.3,.2,.1),T),T)),
    list(pz=rep(.2,T),alpha.star=matrix(rep(c(.2,.5,.1,.1),T),T))
        )

cl=makeCluster(3)
registerDoParallel(cl)

fit=jags.parfit(cl,
                model=EBA.model,
                params=c("pis","alpha","pz","omega","calfcow","bullcow"),
                data=EBA.data,
                inits=EBA.initial,
                n.chains=3,
                n.update=25000,
                n.iter=25000,
                n.thin=1)

stopCluster(cl)
model.fit=summary(fit)
fit.df=rbind(fit[[1]],fit[[2]],fit[[3]])
fit.iter=dim(fit.df)[1]

#################
### OOS Model ###
#################

OOS.model=function(){
```

```
#Priors
#Initial year
pz[1] ~ dbeta(1,1)
pis.temp[1,1:4] <- pis[1,] - pz[1]*omega[1,]
pis.temp[1,5] <- pz[1]

for(j in 1:4){
    alpha.star[1,j] ~ dgamma(.001,.001)
    alpha[1,j] <- alpha.star[1,j]/alpha0[1]
}
alpha0[1] <- sum(alpha.star[,1])

pis[1,] ~ ddirch(alpha[1,])

for(t in 2:T){
    pz[t] ~ dbeta(1,1)
    pis.temp[t,1:4] <- pis[t,] - pz[t]*omega[t,]
    pis.temp[t,5] <- pz[t]

    for(j in 1:4){
        alpha.star[t,j] ~ dgamma(.001,.001)
        alpha[t,j] <- alpha.star[t,j]/alpha0[t]
    }
    alpha0[t]<-sum(alpha.star[t,])

    pis[t,] ~ ddirch(alpha[t,])
}

#Likelihood for counts including unknowns
for(t in 1:T){
    for(i in 1:I[t]){
        Y[t,i,] ~ dmulti(pis.temp[t,],N[t,i])

    }
}

#Likelihood for transect level proportion of calf cow herds
for(t in 1:T){
    for(k in 1:K){
        X[t,k,] ~ dmulti(omega[t,],Nx[t,k])
    }
    omega[t,] ~ ddirch(omega.prior)
}

#Derived parameters
#calf:cow ratio
```

```
    #bull:cow ratio
    for(t in 1:T){
        calfcow[t]<-pis[t,1]/pis[t,2]
        bullcow[t]<-(pis[t,3]+pis[t,4])/pis[t,2]
    }

}#end model

OOS.data=list(pis.temp=matrix(NA,T,J+1),
    pis=matrix(NA,T,J),
    omega=matrix(NA,T,J),
    I=I,
    T=T,
    Y=Y.star,
    N=N.star,
    omega.prior=OOS.omega.prior,
    X=X.oos,
    Nx=Nx.oos,
    K=num.sample,
    pz=rep(NA,T)
    )

OOS.initial=list(
    list(pz=rep(pzset-.05,T),alpha.star=matrix(.25,T,J)),
    list(pz=rep(pzset+.1,T),alpha.star=matrix(rep(c(.4,.3,.2,.1),T),T)),
    list(pz=rep(pzset+.05,T),alpha.star=matrix(rep(c(.2,.5,.1,.1),T),T))
        )

OOS.initial=list(
    list(pz=rep(pzset-.05,T),alpha.star=matrix(.25,T,J)),
    list(pz=rep(pzset+.1,T),alpha.star=matrix(.35,T,J)),
    list(pz=rep(pzset+.05,T),alpha.star=matrix(.45,T,J))
        )

cl=makeCluster(3)
registerDoParallel(cl)

OOS.fit=jags.parfit(cl,
                    model=OOS.model,
                    params=c("pis","alpha","pz","omega","calfcow","bullcow"),
                    data=OOS.data,
                    inits=OOS.initial,
                    n.chains=3,
                    n.update=25000,
                    n.iter=25000,
                    n.thin=1)
stopCluster(cl)
```

```
model.fit.OOS=summary(OOS.fit)

OOS.pis.mean=matrix(model.fit.OOS$statistics[41:60,1],T,J)
OOS.pis.lower=matrix(model.fit.OOS$quantiles[41:60,1],T,J)
OOS.pis.upper=matrix(model.fit.OOS$quantiles[41:60,5],T,J)
OOS.pis.median=matrix(model.fit.OOS$quantiles[41:60,3],T,J)

OOS.fit.df=rbind(OOS.fit[[1]],OOS.fit[[2]],OOS.fit[[3]])
OOS.fit.iter=dim(OOS.fit.df)[1]

##################
### Trim Model ###
##################

#Remove Unknown Column
Y.trim=Y[,,-5]
J.trim=4
N.trim=matrix(NA,T,Imax)

for(t in 1:T){
    N.trim[t,]=apply(Y.trim[t,,],1,sum)
}

TRIM.model=function(){
    for(t in 1:T){
        for(j in 1:J){
            alpha.star[t,j] ~ dgamma(.001,.001)
            alpha[t,j] <- alpha.star[t,j]/alpha0[t]
        }
        alpha0[t]<-sum(alpha.star[t,])
        pis[t,] ~ ddirch(alpha[t,])
    }

    #Likelihood for counts including unknowns
    for(t in 1:T){
        for(i in 1:I[t]){
            Y[t,i,] ~ dmulti(pis[t,],N[t,i])
        }
    }

    #Derived parameters
    #calf:cow ratio
    #bull:cow ratio
    for(t in 1:T){
      calfcow[t]<-pis[t,1]/pis[t,2]
      bullcow[t]<-(pis[t,3]+pis[t,4])/pis[t,2]
```

```
    }

}#end model

data.trim=list(pis=matrix(NA,T,J),
    I=I,
    T=T,
    J=J.trim,
    Y=Y.trim,
    N=N.trim
    )

cl=makeCluster(3)
registerDoParallel(cl)

fit.trim=jags.parfit(cl,
                    model=model.trim,
                    params=c("pis","alpha","calfcow","bullcow"),
                    data=data.trim,
                    n.chains=3,
                    n.update=25000,
                    n.iter=25000,
                    n.thin=1)
stopCluster(cl)

model.fit.trim=summary(fit.trim)

pis.trim.mean=t(rbind(model.fit.trim$statistics[indx.temp.yr1-20,1],
                      model.fit.trim$statistics[indx.temp.yr2-20,1],
                      model.fit.trim$statistics[indx.temp.yr3-20,1],
                      model.fit.trim$statistics[indx.temp.yr4-20,1],
                      model.fit.trim$statistics[indx.temp.yr5-20,1]))

pis.trim.lower=t(rbind(model.fit.trim$quantiles[indx.temp.yr1-20,1],
                       model.fit.trim$quantiles[indx.temp.yr2-20,1],
                       model.fit.trim$quantiles[indx.temp.yr3-20,1],
                       model.fit.trim$quantiles[indx.temp.yr4-20,1],
                       model.fit.trim$quantiles[indx.temp.yr5-20,1]))

pis.trim.upper=t(rbind(model.fit.trim$quantiles[indx.temp.yr1-20,5],
                       model.fit.trim$quantiles[indx.temp.yr2-20,5],
                       model.fit.trim$quantiles[indx.temp.yr3-20,5],
                       model.fit.trim$quantiles[indx.temp.yr4-20,5],
                       model.fit.trim$quantiles[indx.temp.yr5-20,5]))

fit.trim.df=rbind(fit.trim[[1]],fit.trim[[2]],fit.trim[[3]])
fit.trim.iter=dim(fit.trim.df)[1]
```

```
###
### Combined table results, EBA, OOS, and TRIM
###

pis.combined=cbind(EBA.pis.out[,c(1,2,3,5)],
                   OOS.pis.out[,c(1,2,3,5)],
                   pis.out.trim[,5])
names(pis.combined)=c("","Parameter","Truth","EBA","OOS","Trim")
pis.combo=xtable(pis.combined)
digits(pis.combo)=c(0,0,rep(3,8))
print.xtable(pis.combo,
             hline.after=c(0,0,4,8,12,16,20,20),
             include.rownames=FALSE)

EBA_pi2_bias=matrix(NA,nc=T,nr=fit.iter)
EBA_calfcow_bias=matrix(NA,nc=T,nr=fit.iter)
EBA_bullcow_bias=matrix(NA,nc=T,nr=fit.iter)
OOS_pi2_bias=matrix(NA,nc=T,nr=fit.iter)
OOS_calfcow_bias=matrix(NA,nc=T,nr=fit.iter)
OOS_bullcow_bias=matrix(NA,nc=T,nr=fit.iter)
TRIM_pi2_bias=matrix(NA,nc=T,nr=fit.iter)
TRIM_calfcow_bias=matrix(NA,nc=T,nr=fit.iter)
TRIM_bullcow_bias=matrix(NA,nc=T,nr=fit.iter)

#Year 1
for(h in 1:fit.iter){
    EBA_pi2_bias[h,1]=fit.df[h,indx.temp.yr1[2]]-pis.true[1,2]
    EBA_calfcow_bias[h,1]=fit.df[h,26]-calfcow.true[1]
    EBA_bullcow_bias[h,1]=(fit.df[h,61]+fit.df[h,66])/fit.df[h,56]-bullcow.true[illegible]
    TRIM_pi2_bias[h,1]=fit.trim.df[h,indx.temp.yr1[2]-20]-pis.true[1,2]
    TRIM_calfcow_bias[h,1]=fit.trim.df[h,26]-calfcow.true[1]
    TRIM_bullcow_bias[h,1]=fit.trim.df[h,21]-bullcow.true[1]
    OOS_pi2_bias[h,1]=OOS.fit.df[h,indx.temp.yr1[2]]-pis.true[1,2]
    OOS_calfcow_bias[h,1]=OOS.fit.df[h,26]-calfcow.true[1]
    OOS_bullcow_bias[h,1]=OOS.fit.df[h,21]-bullcow.true[1]
}

#Year 2
for(h in 1:fit.iter){
    EBA_pi2_bias[h,2]=fit.df[h,indx.temp.yr2[2]]-pis.true[2,2]
    EBA_bullcow_bias[h,2]=(fit.df[h,62]+fit.df[h,67])/fit.df[h,57]-bullcow.true[illegible]
    EBA_calfcow_bias[h,2]=fit.df[h,27]-calfcow.true[2]
    TRIM_pi2_bias[h,2]=fit.trim.df[h,indx.temp.yr2[2]-20]-pis.true[2,2]
    TRIM_bullcow_bias[h,2]=fit.trim.df[h,22]-bullcow.true[2]
    TRIM_calfcow_bias[h,2]=fit.trim.df[h,27]-calfcow.true[2]
    OOS_pi2_bias[h,2]=OOS.fit.df[h,indx.temp.yr2[2]]-pis.true[2,2]
```

```
    OOS_bullcow_bias[h,2]=OOS.fit.df[h,22]-bullcow.true[2]
    OOS_calfcow_bias[h,2]=OOS.fit.df[h,27]-calfcow.true[2]
}

#Year 3
for(h in 1:fit.iter){
    EBA_pi2_bias[h,3]=fit.df[h,indx.temp.yr3[2]]-pis.true[3,2]
    EBA_bullcow_bias[h,3]=(fit.df[h,63]+fit.df[h,68])/fit.df[h,58]-bullcow.true
    EBA_calfcow_bias[h,3]=fit.df[h,28]-calfcow.true[3]
    TRIM_pi2_bias[h,3]=fit.trim.df[h,indx.temp.yr3[2]-20]-pis.true[3,2]
    TRIM_bullcow_bias[h,3]=fit.trim.df[h,23]-bullcow.true[3]
    TRIM_calfcow_bias[h,3]=fit.trim.df[h,28]-calfcow.true[3]
    OOS_pi2_bias[h,3]=OOS.fit.df[h,indx.temp.yr3[2]]-pis.true[3,2]
    OOS_bullcow_bias[h,3]=OOS.fit.df[h,23]-bullcow.true[3]
    OOS_calfcow_bias[h,3]=OOS.fit.df[h,28]-calfcow.true[3]
}

#Year 4
for(h in 1:fit.iter){
    EBA_pi2_bias[h,4]=fit.df[h,indx.temp.yr4[2]]-pis.true[4,2]
    EBA_bullcow_bias[h,4]=(fit.df[h,64]+fit.df[h,69])/fit.df[h,59]-bullcow.true
    EBA_calfcow_bias[h,4]=fit.df[h,29]-calfcow.true[4]
    TRIM_pi2_bias[h,4]=fit.trim.df[h,indx.temp.yr4[2]-20]-pis.true[4,2]
    TRIM_bullcow_bias[h,4]=fit.trim.df[h,24]-bullcow.true[4]
    TRIM_calfcow_bias[h,4]=fit.trim.df[h,29]-calfcow.true[4]
    OOS_pi2_bias[h,4]=OOS.fit.df[h,indx.temp.yr4[2]]-pis.true[4,2]
    OOS_bullcow_bias[h,4]=OOS.fit.df[h,24]-bullcow.true[4]
    OOS_calfcow_bias[h,4]=OOS.fit.df[h,29]-calfcow.true[4]
}

#Year 5
for(h in 1:fit.iter){
    EBA_pi2_bias[h,5]=fit.df[h,indx.temp.yr5[2]]-pis.true[5,2]
    EBA_bullcow_bias[h,5]=(fit.df[h,65]+fit.df[h,70])/fit.df[h,60]-bullcow.true
    EBA_calfcow_bias[h,5]=fit.df[h,30]-calfcow.true[5]
    TRIM_pi2_bias[h,5]=fit.trim.df[h,indx.temp.yr5[2]-20]-pis.true[5,2]
    TRIM_bullcow_bias[h,5]=fit.trim.df[h,25]-bullcow.true[5]
    TRIM_calfcow_bias[h,5]=fit.trim.df[h,30]-calfcow.true[5]
    OOS_pi2_bias[h,5]=OOS.fit.df[h,indx.temp.yr5[2]]-pis.true[5,2]
    OOS_bullcow_bias[h,5]=OOS.fit.df[h,25]-bullcow.true[5]
    OOS_calfcow_bias[h,5]=OOS.fit.df[h,30]-calfcow.true[5]
}

###
### Return data frames for adult female survival
###
```

```
    EBA_pi2=fit.df[,56:60]
    EBA_pi2stats=model.fit$statistics[56:60,]
    EBA_pi2quants=model.fit$quantiles[56:60,]

    OOS_pi2=OOS.fit.df[,56:60]
    OOS_pi2stats=model.fit.OOS$statistics[56:60,]
    OOS_pi2quants=model.fit.OOS$quantiles[56:60,]

    return(list(pis.true=pis.true,
        bullcow.true=bullcow.true,
        calfcow.true=calfcow.true,
        EBA_pi2_bias=EBA_pi2_bias,
        EBA_pi2=EBA_pi2,
        EBA_pi2stats=EBA_pi2stats,
        EBA_pi2quants=EBA_pi2quants,
        EBA_calfcow_bias=EBA_calfcow_bias,
        EBA_bullcow_bias=EBA_bullcow_bias,
        TRIM_calfcow_bias=trim_calfcow_bias,
        TRIM_bullcow_bias=trim_bullcow_bias,
        TRIM_pi2_bias=TRIM_pi2_bias,
        OOS_calfcow_bias=OOS_calfcow_bias,
        OOS_bullcow_bias=OOS_bullcow_bias,
        OOS_pi2=OOS_pi2,
        OOS_pi2stats=OOS_pi2stats,
        OOS_pi2quants=OOS_pi2quants,
        OOS_pi2_bias=OOS_pi2_bias
        ))
}#end function
```

S4.2 Simulation for Optimal Sub-Sample Size

```
modelrun_ns=function(num.sample.set=8){

    T=5 #number years
    I=c(12,rep(15,4)) #number of surveys in each year
    Imax=max(I) #max number of surveys
    J=4 #number classes

    #set omegas
    omega=matrix(NA,T,J)
    omega[1,]=c(.15,.75,.03,.07)
    omega[2,]=c(.15,.75,.05,.05)
    omega[3,]=c(.2,.71,.03,.04)
    omega[4,]=c(.17,.73,.05,.05)
    omega[5,]=c(.17,.73,.05,.05)

    #Proportion of missing data
    pz = rep(.3,T)

    #Generate true pis
    pis.true = rdirichlet(T,c(30,100,20,20))

    # generate pis.obs
    pis.obs=matrix(NA,T,J+1)
    for(t in 1:T){
        pis.obs[t,] = c((pis.true[t,] - pz[t]*omega[t,]),pz[t])

    }
    pis.obs

    #generate observed matrix of total population counts, using Poisson
    #generate observed proportion in each category, including missing data

    N = matrix(NA,T,Imax)
    Y = array(NA,c(T,Imax,(J+1)))
    #set the number of herds seen within each year within each survey
    K.herd = matrix(NA,T,Imax)
    for(t in 1:T){
        for(i in 1:I[t]){
            N[t,i] = rpois(1,400)
            Y[t,i,]=rmultinom(1,size=N[t,i],pis.obs[t,])
            K.herd[t,i]=rpois(1,10)+1
        }
    }

    #generate X's from the omega's, using a part of the Y's
```

```
X.herd=array(NA,c(T,Imax,max(K.herd,na.rm=TRUE),J))
for(t in 1:T){
    for(i in 1:I[t]){
        herds.sum=sum(Y[t,i,1:J])
        herds.indx=sort(sample(1:herds.sum,K.herd[t,i],replace=FALSE))
        N.herd=c(herds.indx[1],diff(herds.indx))
        for (k in 1:K.herd[t,i]){
            X.herd[t,i,k,]=rmultinom(1,N.herd[k],prob=omega[t,])
        }

    }
}
K=apply(K.herd,1,sum,na.rm=TRUE)
Kmax=max(K)

num.sample=num.sample.set
indx.sample=matrix(NA,T,num.sample)
X.oos=array(NA,c(T,num.sample,J))
Y.star=Y
for(t in 1:T){
    indx.sample[t,]=sort(sample(1:I[t],size=num.sample,replace = TRUE))
    X.oos[t,1,]=X.herd[t,indx.sample[t,1],1,]
    Y.star[t,indx.sample[t,1],1:J]=Y.star[t,indx.sample[t,1],1:J]-X.herd[t,indx
    m=1
    for (h in 2:num.sample){
        if(indx.sample[t,h]==indx.sample[t,h-1]){m=m+1}
        X.oos[t,h,]=X.herd[t,indx.sample[t,h],m,]
        Y.star[t,indx.sample[t,h],1:J]=Y.star[t,indx.sample[t,h],1:J]-X.herd[t,
        m=1
    }
}
Nx.oos=matrix(NA,T,num.sample)
N.star = matrix(NA,T,Imax)
for(t in 1:T){
    Nx.oos[t,]=apply(X.oos[t,,],1,sum)
    N.star[t,] = apply(Y.star[t,,],1,sum)
}
omega.prior=c(1,1,1,1)

#Ensuring the subsample of herds subtracted from Y does not end up in negative
while(length(which(Y.star<0))>0){
  num.sample=10
  indx.sample=matrix(NA,T,num.sample)
  X.oos=array(NA,c(T,num.sample,J))
  Y.star=Y
  for(t in 1:T){
    indx.sample[t,]=sort(sample(1:I[t],size=num.sample,replace = TRUE))
```

```
    X.oos[t,1,]=X.herd[t,indx.sample[t,1],1,]
    Y.star[t,indx.sample[t,1],1:J]=Y.star[t,indx.sample[t,1],1:J]-X.herd[t,indx
    m=1
    for (h in 2:num.sample){
      if(indx.sample[t,h]==indx.sample[t,h-1]){m=m+1}
      X.oos[t,h,]=X.herd[t,indx.sample[t,h],m,]
      Y.star[t,indx.sample[t,h],1:J]=Y.star[t,indx.sample[t,h],1:J]-X.herd[t,in
      m=1
    }
  }
  Nx.oos=matrix(NA,T,num.sample)
  N.star = matrix(NA,T,Imax)
  for(t in 1:T){
    Nx.oos[t,]=apply(X.oos[t,,],1,sum)
    N.star[t,] = apply(Y.star[t,,],1,sum)
  }
}

######################
### Jags Model OOS ###
######################

OOS.model=function(){

    #Priors
    #Initial year
    pz[1] ~ dbeta(1,1)
    pis.temp[1,1:4] <- pis[1,] - pz[1]*omega[1,]
    pis.temp[1,5] <- pz[1]

    for(j in 1:4){
        alpha.star[1,j] ~ dgamma(.001,.001)
        alpha[1,j] <- alpha.star[1,j]/alpha0[1]
    }
    alpha0[1] <- sum(alpha.star[,1])

    pis[1,] ~ ddirch(alpha[1,])

    for(t in 2:T){
        pz[t] ~ dbeta(1,1)
        pis.temp[t,1:4] <- pis[t,] - pz[t]*omega[t,]
        pis.temp[t,5] <- pz[t]

        for(j in 1:4){
            alpha.star[t,j] ~ dgamma(.001,.001)
            alpha[t,j] <- alpha.star[t,j]/alpha0[t]
        }
```

```
        alpha0[t]<-sum(alpha.star[t,])

        pis[t,] ~ ddirch(alpha[t,])
    }

    #Likelihood for counts including unknowns
    for(t in 1:T){
        for(i in 1:I[t]){
            Y[t,i,] ~ dmulti(pis.temp[t,],N[t,i])
        }
    }

    #Likelihood for transect level proportion of calf cow herds
    for(t in 1:T){
        for(k in 1:K){
            X[t,k,] ~ dmulti(omega[t,],Nx[t,k])
        }
        omega[t,] ~ ddirch(omega.prior)
    }

    #Derived parameters
    #calf:cow ratio
    #bull:cow ratio
    for(t in 1:T){
        calfcow[t]<-pis[t,1]/pis[t,2]
        bullcow[t]<-(pis[t,3]+pis[t,4])/pis[t,2]
    }
}#end model

OOS.data=list(pis.temp=matrix(NA,T,J+1),
    pis=matrix(NA,T,J),
    omega=matrix(NA,T,J),
    I=I,
    T=T,
    Y=Y.star,
    N=N.star,
    omega.prior=omega.prior,
    X=X.oos,
    Nx=Nx.oos,
    K=num.sample,
    pz=rep(NA,T)
    )

OOS.initial=list(
    list(pz=rep(.1,T),alpha.star=matrix(.25,T,J)),
    list(pz=rep(.3,T),alpha.star=matrix(rep(c(.4,.3,.2,.1),T),T)),
    list(pz=rep(.2,T),alpha.star=matrix(rep(c(.2,.5,.1,.1),T),T))
```

```
        )

cl=makeCluster(3)
registerDoParallel(cl)
OOS.fit=jags.parfit(cl,
                    model=OOS.model,
                    params=c("pis","alpha","pz","omega","calfcow","bullcow"),
                    data=OOS.data,
                    inits=OOS.initial,
                    n.chains=3,
                    n.update=25000,
                    n.iter=25000,
                    n.thin=1)

model.fit.OOS=summary(OOS.fit)
OOS.fit.df=rbind(OOS.fit[[1]],OOS.fit[[2]],OOS.fit[[3]])
OOS.fit.iter=dim(OOS.fit.df)[1]

#######################
### Jags Model TRIM ###
#######################

### Fit the model, without unknown category
Y.TRIM=Y[,,-5]
J.TRIM=4
N.TRIM=matrix(NA,T,Imax)

for(t in 1:T){
    N.TRIM[t,]=apply(Y.TRIM[t,,],1,sum)
}

model.TRIM=function(){
    for(t in 1:T){
        for(j in 1:J){
            alpha.star[t,j] ~ dgamma(.001,.001)
            alpha[t,j] <- alpha.star[t,j]/alpha0[t]
        }
        alpha0[t]<-sum(alpha.star[t,])
        pis[t,] ~ ddirch(alpha[t,])
    }

    #Likelihood for counts including unknowns
    for(t in 1:T){
        for(i in 1:I[t]){
            Y[t,i,] ~ dmulti(pis[t,],N[t,i])
        }
    }
```

```
    #Derived parameters
    #calf:cow ratio
    #bull:cow ratio
    for(t in 1:T){
        calfcow[t]<-pis[t,1]/pis[t,2]
        bullcow[t]<-(pis[t,3]+pis[t,4])/pis[t,2]
    }
}#end model

data.TRIM=list(pis=matrix(NA,T,J),
    I=I,
    T=T,
    J=J.TRIM,
    Y=Y.TRIM,
    N=N.TRIM
    )

cl=makeCluster(3)
registerDoParallel(cl)

fit.TRIM=jags.parfit(cl,
                     model=model.TRIM,
                     params=c("pis","alpha","calfcow","bullcow"),
                     data=data.TRIM,
                     n.chains=3,
                     n.update=25000,
                     n.iter=25000,
                     n.thin=1)

model.fit.TRIM=summary(fit.TRIM)

pis.TRIM.mean=t(rbind(model.fit.TRIM$statistics[indx.temp.yr1-20,1],
                      model.fit.TRIM$statistics[indx.temp.yr2-20,1],
                      model.fit.TRIM$statistics[indx.temp.yr3-20,1],
                      model.fit.TRIM$statistics[indx.temp.yr4-20,1],
                      model.fit.TRIM$statistics[indx.temp.yr5-20,1]))

pis.TRIM.lower=t(rbind(model.fit.TRIM$quantiles[indx.temp.yr1-20,1],
                       model.fit.TRIM$quantiles[indx.temp.yr2-20,1],
                       model.fit.TRIM$quantiles[indx.temp.yr3-20,1],
                       model.fit.TRIM$quantiles[indx.temp.yr4-20,1],
                       model.fit.TRIM$quantiles[indx.temp.yr5-20,1]))

pis.TRIM.upper=t(rbind(model.fit.TRIM$quantiles[indx.temp.yr1-20,5],
                       model.fit.TRIM$quantiles[indx.temp.yr2-20,5],
                       model.fit.TRIM$quantiles[indx.temp.yr3-20,5],
```

```
                        model.fit.TRIM$quantiles[indx.temp.yr4-20,5],
                        model.fit.TRIM$quantiles[indx.temp.yr5-20,5]))

fit.TRIM.df=rbind(fit.TRIM[[1]],fit.TRIM[[2]],fit.TRIM[[3]])
fit.TRIM.iter=dim(fit.TRIM.df)[1]

###
### Combined table results, full and TRIM
###

pis.combined=cbind(OOS.pis.out[,c(1,2,3,5)],
                   pis.out.TRIM[,5])
names(pis.combined)=c("","Parameter","Truth","OOS","TRIM")
pis.combo=xtable(pis.combined)
digits(pis.combo)=c(0,0,3,3,3,3)
print.xtable(pis.combo,
             hline.after=c(0,0,4,8,12,16,20,20),
             include.rownames=FALSE)

OOS_pi2_bias=matrix(NA,nc=T,nr=OOS.fit.iter)
OOS_calfcow_bias=matrix(NA,nc=T,nr=OOS.fit.iter)
OOS_bullcow_bias=matrix(NA,nc=T,nr=OOS.fit.iter)
TRIM_pi2_bias=matrix(NA,nc=T,nr=OOS.fit.iter)
TRIM_calfcow_bias=matrix(NA,nc=T,nr=OOS.fit.iter)
TRIM_bullcow_bias=matrix(NA,nc=T,nr=OOS.fit.iter)

for(h in 1:OOS.fit.iter){
    OOS_pi2_bias[h,1]=OOS.fit.df[h,indx.temp.yr1[2]]-pis.true[1,2]
    OOS_calfcow_bias[h,1]=OOS.fit.df[h,26]-calfcow.true[1]
    OOS_bullcow_bias[h,1]=OOS.fit.df[h,21]-bullcow.true[1]
    TRIM_calfcow_bias[h,1]=fit.TRIM.df[h,26]-calfcow.true[1]
    TRIM_bullcow_bias[h,1]=fit.TRIM.df[h,21]-bullcow.true[1]
    TRIM_pi2_bias[h,1]=fit.TRIM.df[h,indx.temp.yr1[2]-20]-pis.true[1,2]
}

for(h in 1:OOS.fit.iter){
    OOS_pi2_bias[h,2]=OOS.fit.df[h,indx.temp.yr2[2]]-pis.true[2,2]
    OOS_bullcow_bias[h,2]=OOS.fit.df[h,22]-bullcow.true[2]
    OOS_calfcow_bias[h,2]=OOS.fit.df[h,27]-calfcow.true[2]
    TRIM_pi2_bias[h,2]=fit.TRIM.df[h,indx.temp.yr2[2]-20]-pis.true[2,2]
    TRIM_bullcow_bias[h,2]=fit.TRIM.df[h,22]-bullcow.true[2]
    TRIM_calfcow_bias[h,2]=fit.TRIM.df[h,27]-calfcow.true[2]
}

for(h in 1:OOS.fit.iter){
    OOS_pi2_bias[h,3]=OOS.fit.df[h,indx.temp.yr3[2]]-pis.true[3,2]
    OOS_bullcow_bias[h,3]=OOS.fit.df[h,23]-bullcow.true[3]
```

```
        OOS_calfcow_bias[h,3]=OOS.fit.df[h,28]-calfcow.true[3]
        TRIM_pi2_bias[h,3]=fit.TRIM.df[h,indx.temp.yr3[2]-20]-pis.true[3,2]
        TRIM_bullcow_bias[h,3]=fit.TRIM.df[h,23]-bullcow.true[3]
        TRIM_calfcow_bias[h,3]=fit.TRIM.df[h,28]-calfcow.true[3]
    }

    for(h in 1:OOS.fit.iter){
        OOS_pi2_bias[h,4]=OOS.fit.df[h,indx.temp.yr4[2]]-pis.true[4,2]
        OOS_bullcow_bias[h,4]=OOS.fit.df[h,24]-bullcow.true[4]
        OOS_calfcow_bias[h,4]=OOS.fit.df[h,29]-calfcow.true[4]
        TRIM_pi2_bias[h,4]=fit.TRIM.df[h,indx.temp.yr4[2]-20]-pis.true[4,2]
        TRIM_bullcow_bias[h,4]=fit.TRIM.df[h,24]-bullcow.true[4]
        TRIM_calfcow_bias[h,4]=fit.TRIM.df[h,29]-calfcow.true[4]
    }

    for(h in 1:OOS.fit.iter){
        OOS_pi2_bias[h,5]=OOS.fit.df[h,indx.temp.yr5[2]]-pis.true[5,2]
        OOS_bullcow_bias[h,5]=OOS.fit.df[h,25]-bullcow.true[5]
        OOS_calfcow_bias[h,5]=OOS.fit.df[h,30]-calfcow.true[5]
        TRIM_pi2_bias[h,5]=fit.TRIM.df[h,indx.temp.yr5[2]-20]-pis.true[5,2]
        TRIM_bullcow_bias[h,5]=fit.TRIM.df[h,25]-bullcow.true[5]
        TRIM_calfcow_bias[h,5]=fit.TRIM.df[h,30]-calfcow.true[5]
    }

    ###
    ### Return data frames for adult female survival
    ###

    OOS_pi2=OOS.fit.df[,56:60]
    OOS_pi2stats=model.fit.OOS$statistics[56:60,]
    OOS_pi2quants=model.fit.OOS$quantiles[56:60,]

    return(list(OOS_pi2_bias=OOS_pi2_bias,
                TRIM_pi2_bias=TRIM_pi2_bias,
                OOS_pi2=OOS_pi2,
                OOS_pi2stats=OOS_pi2stats,
                OOS_pi2quants=OOS_pi2quants,
                pis.true=pis.true,
                OOS_calfcow_bias=OOS_calfcow_bias,
                OOS_bullcow_bias=OOS_bullcow_bias,
                bullcow.true=bullcow.true,
                calfcow.true=calfcow.true,
                TRIM_calfcow_bias=TRIM_calfcow_bias,
                TRIM_bullcow_bias=TRIM_bullcow_bias))
}#end function
```
