# Supplementary material for: A hierarchical Bayesian approach for handling missing classification data
Source: Ecol Evol. 2019 Mar 2;9(6):3130–40. doi: 10.1002/ece3.4927 (PMC6434567; doi:10.1002/ece3.4927)
Supplement: Supplementary file 3 [file ECE3-9-3130-s003.pdf]

Appendix S3 - Simulation Plots

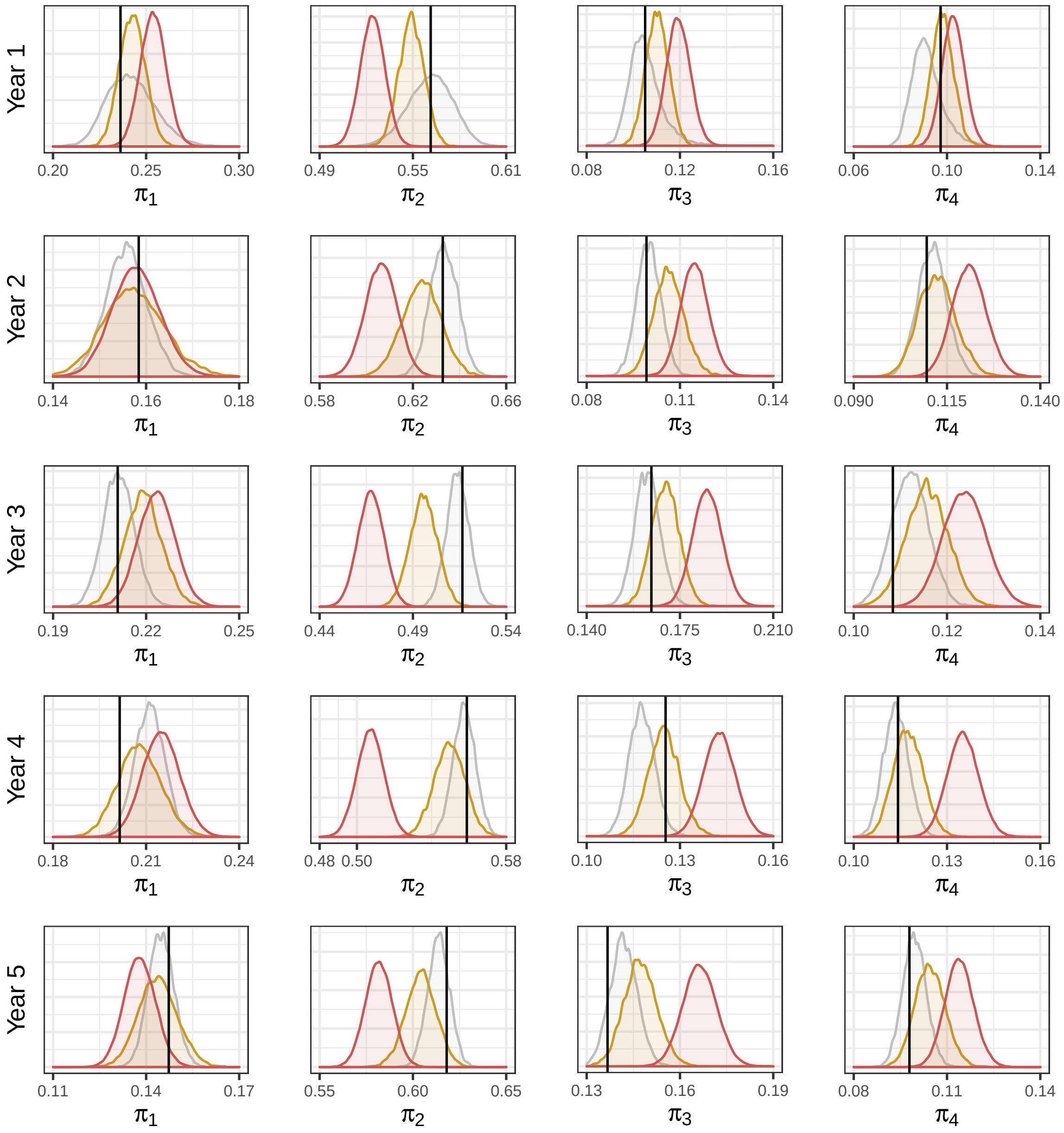

Figure S1: The densities of the marginal posterior distributions for the proportions of each simulated stage/sex classes including juveniles (π_1), yearling and adult females (π_2), yearling males (π_3), and adult males (π_4) for all five years of the simulation, using the empirical Bayesian approach (grey), out of sample approach (yellow), and ignoring the unclassified data (red). The vertical black line is the true value of the parameter used to generate the data.

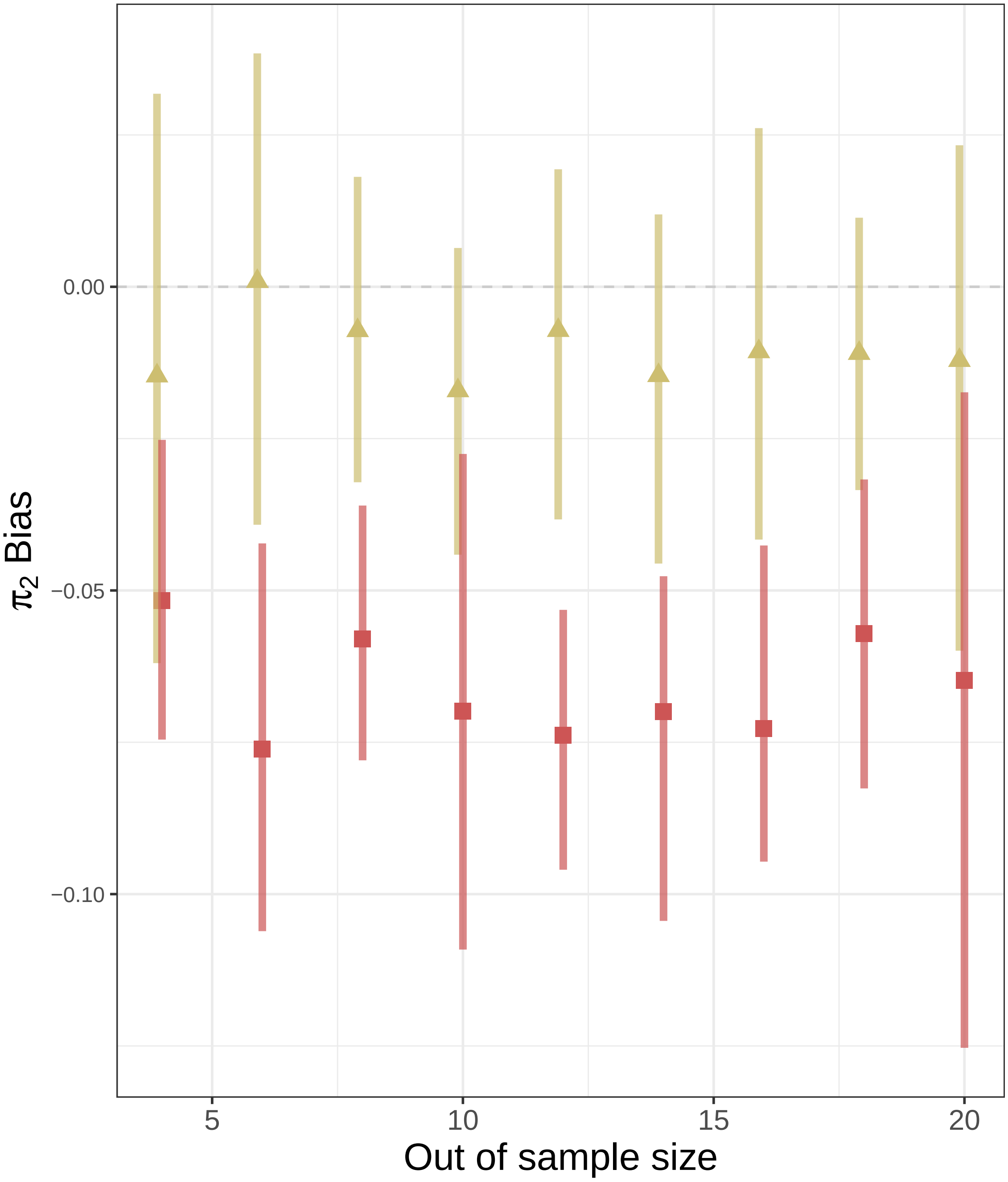

Figure S2: The posterior distributions of the difference between the generated proportion of adult and yearling females π_2 and the true value for the out-of-sample approach (yellow triangles), and ignoring the unclassified data (red squares), for a constant proportion of missing unclassified data (p_z), with increasing the random sample sizes used to estimate the distributions of unknowns. The vertical bars represent the 95% equal-tailed Bayesian credible intervals. The horizontal dashed line indicates no bias.
