# Supplementary material for: A hierarchical Bayesian approach for handling missing classification data
Source: Ecol Evol. 2019 Mar 2;9(6):3130–40. doi: 10.1002/ece3.4927 (PMC6434567; doi:10.1002/ece3.4927)
Supplement: Supplementary file 4 [file ECE3-9-3130-s004.pdf]

Appendix S4 - Case study Results

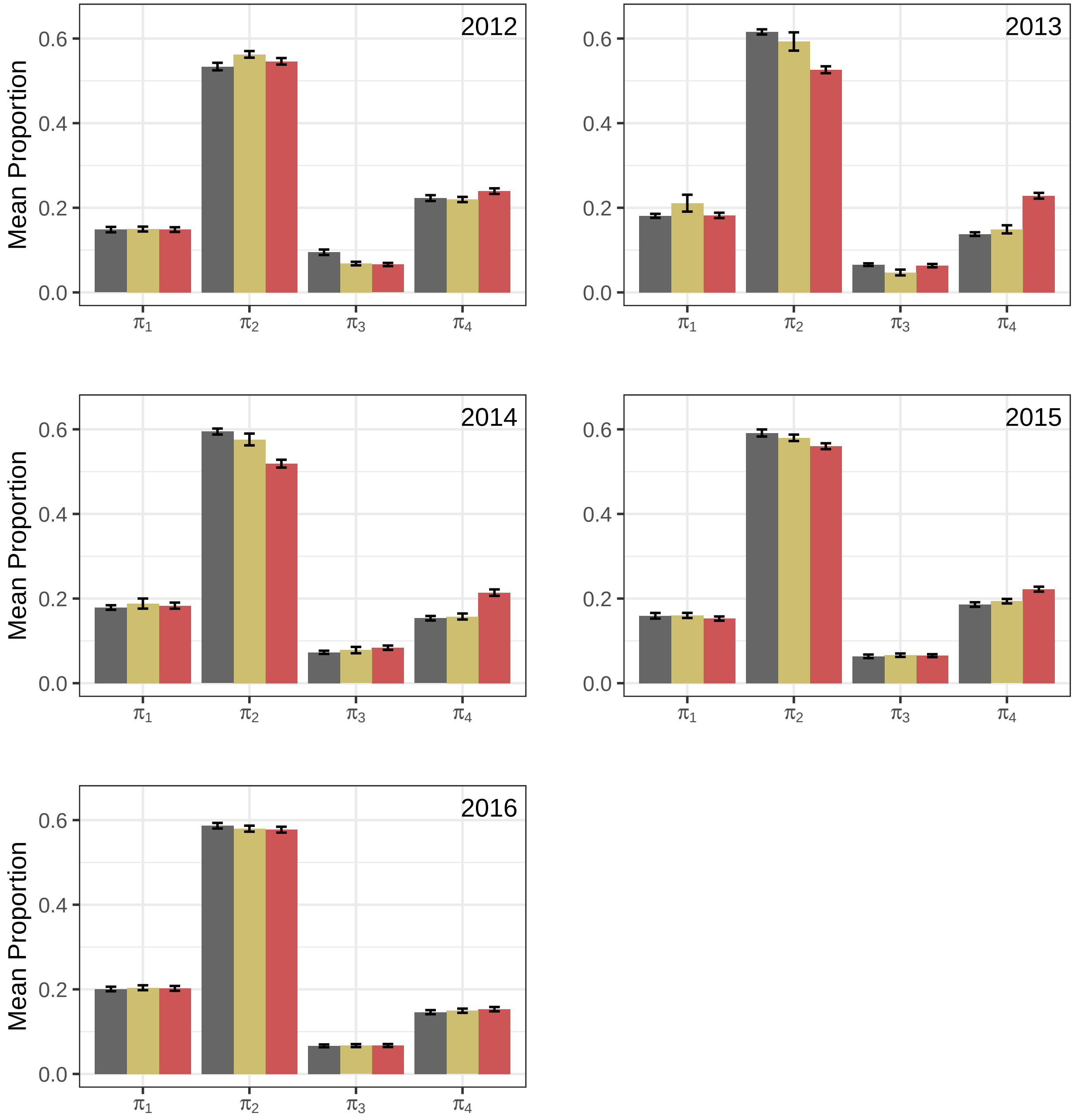

Figure S1: The means of the proportions of sex and stage classes of elk in Rocky Mountain National Park ($\boldsymbol{\pi}_t$) in the tth year for the empirical Bayes model (black), out-of-sample moel (yellow), and trim model ignoring the unclassified data (red), with $\pm$ 1 standard deviation error bars (black lines).

Combined Parameter Table

Table S1: Medians of the posterior distributions of the proportions of each class ($\boldsymbol{\pi}$) from 2012 through 2016 for elk in Rocky Mountain National Park derived from three models including the empirical Bayes approach (EBA), out-of-sample (OOS), and ignoring (Trim) approaches.

Year	Parameter	EBA Median	OOS Median	Trim Median
	π_1	0.148	0.152	0.148
2012	π_2	0.534	0.561	0.546
	π_3	0.095	0.065	0.066
	π_4	0.223	0.222	0.239
	π_1	0.181	0.183	0.182
2013	π_2	0.616	0.615	0.526
	π_3	0.066	0.054	0.063
	π_4	0.138	0.147	0.228
	π_1	0.179	0.188	0.183
2014	π_2	0.595	0.576	0.519
	π_3	0.073	0.078	0.084
	π_4	0.153	0.158	0.214
	π_1	0.159	0.153	0.153
2015	π_2	0.592	0.597	0.560
	π_3	0.063	0.055	0.065
	π_4	0.186	0.194	0.222
	π_1	0.201	0.203	0.202
2016	π_2	0.587	0.577	0.577
	π_3	0.066	0.068	0.067
	π_4	0.146	0.151	0.153

Parameter Tables

Parameter tables consist of results using the observed data for multiple years of parameters with associated equal-tailed Bayesian credible intervals for all three models.

Table S2: Summary statistics of the marginal posterior distributions of the proportions of all classes ($\boldsymbol{\pi}$) from 2012 through 2016 for elk in Rocky Mountain National Park using the empirical Bayes approach.

Year	Parameter	Mean	Median	SD	.025	.975
	π_1	0.148	0.148	0.006	0.136	0.161
2012	π_2	0.534	0.534	0.009	0.516	0.551
	π_3	0.095	0.095	0.006	0.083	0.108
	π_4	0.223	0.223	0.007	0.210	0.237
	π_1	0.181	0.181	0.005	0.172	0.190
2013	π_2	0.616	0.616	0.006	0.604	0.627
	π_3	0.066	0.066	0.003	0.060	0.072
	π_4	0.138	0.138	0.004	0.130	0.146
	π_1	0.179	0.179	0.005	0.168	0.189
2014	π_2	0.595	0.595	0.007	0.581	0.608
	π_3	0.073	0.073	0.004	0.066	0.080
	π_4	0.153	0.153	0.005	0.143	0.164
	π_1	0.159	0.159	0.007	0.147	0.173
2015	π_2	0.591	0.591	0.008	0.575	0.608
	π_3	0.063	0.063	0.004	0.056	0.072
	π_4	0.186	0.186	0.005	0.176	0.197
	π_1	0.201	0.201	0.005	0.190	0.211
2016	π_2	0.587	0.587	0.007	0.574	0.600
	π_3	0.066	0.066	0.003	0.060	0.073
	π_4	0.146	0.146	0.005	0.137	0.156

Table S3: Summary statistics of the marginal posterior distributions of the proportions of all classes ($\boldsymbol{\pi}$) from 2012 through 2016 for elk in Rocky Mountain National Park using the out-of-sample approach.

	Parameter	Mean	Median	SD	.025	.975
	π_1	0.150	0.150	0.006	0.139	0.161
2012	π_2	0.563	0.563	0.008	0.547	0.578
	π_3	0.068	0.068	0.004	0.060	0.077
	π_4	0.220	0.220	0.006	0.208	0.232
	π_1	0.211	0.210	0.020	0.175	0.252
2013	π_2	0.593	0.594	0.022	0.548	0.634
	π_3	0.047	0.046	0.007	0.037	0.063
	π_4	0.149	0.148	0.010	0.134	0.171
	π_1	0.188	0.188	0.012	0.166	0.212
2014	π_2	0.576	0.576	0.014	0.548	0.603
	π_3	0.078	0.078	0.007	0.065	0.094
	π_4	0.158	0.157	0.007	0.145	0.172
	π_1	0.160	0.160	0.006	0.149	0.172
2015	π_2	0.580	0.580	0.008	0.565	0.595
	π_3	0.066	0.066	0.004	0.059	0.074
	π_4	0.194	0.194	0.005	0.184	0.204
	π_1	0.204	0.204	0.006	0.193	0.215
2016	π_2	0.580	0.580	0.007	0.566	0.594
	π_3	0.067	0.067	0.004	0.060	0.074
	π_4	0.149	0.149	0.005	0.140	0.159

Table S4: Summary statistics of the marginal posterior distributions of the proportions of all classes ($\boldsymbol{\pi}$) from 2012 through 2016 for elk in Rocky Mountain National Park using the ignoring (trim) approach.

	Parameter	Mean	Median	SD	.025	.975
2012	π_1	0.149	0.148	0.005	0.138	0.159
	π_2	0.546	0.546	0.008	0.531	0.561
	π_3	0.066	0.066	0.004	0.059	0.074
	π_4	0.239	0.239	0.007	0.227	0.252
2013	π_1	0.182	0.182	0.006	0.170	0.194
	π_2	0.526	0.526	0.008	0.510	0.542
	π_3	0.063	0.063	0.004	0.056	0.071
	π_4	0.228	0.228	0.007	0.215	0.242
2014	π_1	0.183	0.183	0.007	0.169	0.197
	π_2	0.519	0.519	0.009	0.501	0.537
	π_3	0.084	0.084	0.005	0.074	0.094
	π_4	0.214	0.214	0.008	0.199	0.229
2015	π_1	0.153	0.153	0.005	0.143	0.163
	π_2	0.560	0.560	0.007	0.547	0.574
	π_3	0.065	0.065	0.003	0.058	0.072
	π_4	0.222	0.222	0.006	0.211	0.234
2016	π_1	0.202	0.202	0.006	0.191	0.214
	π_2	0.577	0.577	0.007	0.564	0.591
	π_3	0.067	0.067	0.004	0.060	0.074
	π_4	0.153	0.153	0.005	0.143	0.163
